# Exercise–nutrition–myokine–mitochondrial interactions in immune aging: an immunometabolic framework for host defense

**DOI:** 10.3389/fimmu.2026.1987314

**Published:** 2026-10-30

**Authors:** Ran Peng, Yan Pan, Junpeng Pang, Rui Xu, Yayi Jiang

**Affiliations:** 1 Wuhan Sports University, Wuhan, China; 2 Department of Electronic Engineering, Tsinghua University, Beijing, China; 3 Graduate School of Trainers, Belarusian State University of Physical Culture, Minsk, Belarus

**Keywords:** exercise, host defense, immune aging, immunometabolism, mitochondrial adaptation, myokines, nutrition

## Abstract

Exercise is increasingly recognized as a systemic modulator of immune and metabolic physiology, with potential relevance to immune aging, chronic low-grade inflammation, and host defense. This structured narrative review integrates evidence on the interplay among exercise, skeletal-muscle-derived signaling, mitochondrial adaptation, and nutritional context to develop a conceptual framework for immunometabolic reprogramming during immune aging. Literature was identified through searches of PubMed/MEDLINE, Web of Science, Scopus, and Google Scholar using combinations of terms related to exercise, myokines/exerkines, mitochondria, nutrition, immunosenescence, inflammaging, and host defense. We synthesize evidence across human, animal, and cellular studies, while distinguishing direct human findings from associative and preclinical mechanistic evidence. Particular attention is given to exercise-induced myokine signaling, mitochondrial biogenesis and function, metabolic flexibility, redox signaling, and mitochondrial quality control, as well as the potential modifying effects of macronutrients, amino acids, fatty acids, micronutrients, and dietary bioactive compounds. The review further considers how exercise and nutritional context may influence features of immunosenescence and inflammaging and examines their potential relevance to vaccine responses and infection-related host defense. Importantly, changes in circulating biomarkers or immune-cell phenotypes are not assumed to represent improved immune competence or clinical protection. Instead, the available evidence is interpreted according to exercise modality and dose, training status, age, sex, nutritional and metabolic context, and the level of biological evidence. Overall, this framework highlights the potential for exercise–nutrition interactions to shape immune–metabolic communication while emphasizing that several proposed myokine–mitochondrial–immune links remain associative or mechanistic hypotheses requiring prospective human investigation.

## 1 Introduction

Exercise is increasingly recognized not only as a means of improving physical fitness and metabolic health, but also as a systemic biological stimulus that can shape how metabolic, endocrine, and immune systems communicate. Skeletal muscle is central to this framework, since its contraction coincides with the release of a wide array of signaling molecules, collectively termed myokines or, more broadly, exerkines. However, exercise-induced inter-organ communication is not restricted to skeletal muscle; exerkines may also originate from or be influenced by adipose tissue, liver, bone, vascular endothelium, pancreas, and other tissues, making circulating exercise-responsive factors biologically heterogeneous rather than exclusively muscle-derived (1, 2). Moreover, muscle expression of a candidate myokine, its secretion into the circulation, its measurable circulating concentration, receptor engagement in a target tissue, and a demonstrated functional immune effect represent distinct biological steps and should not be considered interchangeable (2). These mediators may help coordinate communication between skeletal muscle and distant tissues, and they have been implicated in metabolic control, inflammatory signaling, and immune activity (3–5). Accordingly, exercise-driven signaling has become an important element of the wider intersection between metabolism and immunity. Importantly, increased expression of a mediator within skeletal muscle, its secretion from muscle, an increase in its circulating concentration, receptor engagement in immune cells, and a demonstrable functional immune effect represent biologically distinct steps. Therefore, these levels of evidence should not be considered interchangeable when interpreting exercise-associated myokine signaling.

The framework of immunometabolism offers a valuable lens for interpreting this intersection. During immune-cell activation, differentiation, migration, and the resolution of immune responses, immune cells make major metabolic adjustments, and metabolic pathways can shape immune-cell phenotype and function, rather than merely supplying cellular energy (6, 7). Exercise is a physiological disturbance that can alter both systemic and cellular metabolism. Studies in humans have found that leukocyte energy metabolism shifts after acute exercise or exercise training; however, the size and direction of these changes seem to depend on the immune-cell subset, the features of the exercise, the individual’s training status, and participant characteristics (8). From an immunometabolic perspective, immune-cell activation, differentiation, migration, and resolution require coordinated metabolic adaptations. Exercise itself represents a physiological perturbation that can transiently alter leukocyte trafficking, metabolism, and inflammatory signaling. Importantly, these responses should be interpreted in relation to whether exercise is acute or chronic and according to exercise modality, intensity, duration, training status, age, sex, and nutritional state, because these factors can substantially modify the magnitude and direction of exercise-associated immune responses (9). Accordingly, exercise-related immune effects may reflect coordinated metabolic remodeling rather than a uniform change in circulating inflammatory markers.

Mitochondria are especially pertinent to this framework because they coordinate energy generation, redox signaling, cellular stress responses, and metabolic adaptation. In immune cells, mitochondrial metabolism supports functional specialization and drives immune activation, whereas mitochondrial dysfunction has been associated with changes in inflammatory regulation and cellular aging (10, 11). Exercise serves as a strong trigger for mitochondrial adaptation in skeletal muscle, and a growing body of evidence suggests that physical activity may also affect mitochondrial traits throughout the lifespan (12, 13). A key question is whether, and to what degree, adaptations in skeletal muscle mitochondria translate into functionally significant alterations in immune-cell metabolism. This distinction is critical because increased expression of PGC-1α or mitochondrial proteins does not necessarily establish increased mitochondrial abundance, completed mitochondrial turnover, or improved mitochondrial function. Moreover, evidence that exercise alters mitochondrial biogenesis or quality control in skeletal muscle does not by itself demonstrate a corresponding mitochondrial adaptation in circulating or tissue-resident immune cells. Such relationships should therefore be distinguished as direct human evidence, associative evidence, preclinical mechanistic evidence, or hypothesized integrative links, according to the available data.

Aging is accompanied by substantial remodeling of both innate and adaptive immunity. Features of immunosenescence include thymic involution, altered naïve-to-memory T-cell composition, accumulation of late-differentiated or senescent T-cell populations, changes in B-cell and natural killer (NK)-cell compartments, and broader alterations in immune-cell metabolism and hematopoiesis (14). In parallel, inflammaging refers primarily to a persistent, low-grade, systemic inflammatory state associated with aging and should not be treated as synonymous with immunosenescence, although the two processes are biologically interconnected (14, 15). Regular physical activity has been associated with favorable changes in several immune and inflammatory markers in older adults, but changes in circulating inflammatory biomarkers or immune-cell phenotypes should not automatically be interpreted as reversal of immunosenescence or restoration of immune competence (9, 16). Immune aging entails extensive restructuring of both innate and adaptive immune compartments and may involve changes in immune-cell composition, reduced responsiveness to certain immune challenges, and ongoing low-grade inflammatory activity often referred to as inflammaging (17). These developments have been linked to diminished immune resilience and altered reactions to infectious threats and other environmental challenges. Findings from exercise research indicate that regular physical activity may be associated with more favorable patterns of multiple cellular and inflammatory markers related to immunosenescence; however, results vary across studies and do not demonstrate that exercise can consistently reverse age-related immune alterations (18). Changes in inflammatory biomarkers should therefore not be interpreted automatically as evidence of reversal of cellular immune aging or improved host defense. The metabolic condition of immune cells, mitochondrial integrity, and the availability of metabolic substrates may be key determinants underlying this variability. Sex and reproductive status may further modify immune aging and exercise-associated responses. Sex-related differences in innate and adaptive immunity are influenced by sex chromosomes and sex hormones and may change across the life course, including during menopause and endocrine aging (19, 20). Accordingly, the interpretation of exercise–immune interactions should consider sex composition, hormonal status, body composition, and, where available, sex-stratified analyses rather than assuming uniform responses across men and women.

Nutritional status is another potentially significant factor that can influence exercise-induced immunometabolic responses. Nutrients supply the substrates and cofactors needed for energy metabolism, biosynthesis, redox regulation, and the activity of immune cells. Aging may also change how nutritional intake relates to immune function, which makes the nutritional context especially important when interpreting immune responses in older adults (21). At the same time, the body of evidence on combined exercise and nutritional interventions is still less established than the evidence addressing either component on its own. More recent findings have pointed to the possibility that dietary modifications that affect metabolic pathways could alter exercise-associated immunometabolic adaptations, although the clinical importance of such combined approaches remains unclear (22). Within the exercise–immune interface, nutritional status may be conceptualized through at least three partially distinct roles: as a direct regulator of immune-cell metabolism, as a modifier of exercise-induced signaling and adaptation, and as a determinant of substrate availability and recovery. These effects may depend on energy availability, protein and amino-acid intake, micronutrient status, metabolic health, and dietary composition (23, 24). The gut microbiome may also represent an additional mechanistic interface linking diet, exercise, metabolism, and immune regulation, although microbiome-mediated effects are not assumed to account for all exercise–nutrition interactions (24).

The connection between exercise and host defense is similarly intricate. Regular physical activity has generally been linked to elements of immune surveillance and overall health, while the immune effects of extended, high-volume, or strenuous exercise are still harder to clarify. The present evidence does not support a straightforward framework in which exercise automatically suppresses immunity; instead, vulnerability to infection appears to depend on several interacting factors, such as exercise dose, recovery, nutritional status, psychological stress, sleep, environmental exposure, and individual characteristics (25). This context-dependent association is especially important when evaluating the possible role of exercise-induced immunometabolic remodeling in antimicrobial and antiviral host defense. Importantly, molecular and cellular responses should be distinguished from clinically meaningful host-defense outcomes. Changes in cytokines, immune-cell phenotype, mitochondrial respiration, or other metabolic biomarkers may provide mechanistic information but do not necessarily demonstrate reduced infection incidence, severity, or duration, enhanced vaccine effectiveness, or improved clinical outcomes (16, 26). Direct evidence for these clinically relevant endpoints, particularly in older adults with established immune aging or in immunologically vulnerable populations, remains comparatively limited (16, 27).

Despite considerable advances in exercise immunology, these topics are frequently studied in isolation. Myokine biology, mitochondrial adaptation, immune-cell metabolism, nutrition, immune aging, and host defense are interrelated processes; however, a unified framework that links them is still not fully established. Accordingly, this narrative review consolidates current evidence regarding exercise-induced immunometabolic reprogramming, with particular focus on the interactions among skeletal muscle–derived myokines, mitochondrial adaptation, and nutritional signals. We also examine how these connected processes may relate to immune aging and host defense, while identifying areas where the available evidence is heterogeneous or insufficient for determining causality. Rather than proposing a new molecular pathway, the conceptual contribution of this review is to integrate research domains that are often considered separately and to organize their relationships according to exercise dose and modality, nutritional context, biological level, and strength of evidence. In particular, the review distinguishes acute exercise responses from chronic training adaptations and separates direct human observations from associative findings, preclinical mechanistic evidence, and hypothesized cross-system connections.

Existing reviews have extensively examined exercise and immunity, myokine signaling, mitochondrial adaptation, nutritional modulation, immunosenescence, inflammaging, and exercise-associated host defense as individual or partially overlapping topics. The present review extends this literature by examining these domains within an integrated exercise–nutrition–myokine–mitochondrial–immune-aging framework. This framework emphasizes that exercise-induced immunometabolic remodeling is likely to be conditional rather than uniform, with responses shaped by exercise dose, training status, age, sex, nutritional and metabolic context, and the biological level at which an outcome is measured. It further highlights an important translational distinction between molecular or cellular adaptation and demonstrable immune competence or clinical host defense. By explicitly identifying where mechanistic links are directly demonstrated, indirectly inferred, or primarily hypothetical, the review aims to provide a structured basis for interpreting current evidence and identifying priorities for prospective human research rather than implying that the proposed pathways are already clinically established.

## 2 Literature search strategy and methodology

This article was conducted as a structured narrative review designed to identify, critically organize, and conceptually integrate evidence concerning exercise-induced immunometabolic reprogramming, with particular emphasis on the interactions among skeletal-muscle-derived signaling, myokines/exerkines, mitochondrial adaptation, nutritional regulation, immune aging, inflammation, and host defense. The methodological approach was structured to improve transparency and reproducibility while remaining consistent with the scope of a narrative rather than a systematic review. Accordingly, the objective was not to identify every potentially eligible publication or to generate pooled effect estimates, but to synthesize mechanistically and clinically informative evidence across complementary biological levels. The reporting framework was informed by principles for high-quality narrative reviews, including explicit description of the literature search, evidence level, and relevant endpoints (28).

Searches were conducted in PubMed/MEDLINE, Web of Science Core Collection, Scopus, and Google Scholar. The final search was conducted in July 2026, covering publications from database inception through 31 July 2026. Studies were eligible for consideration when an English-language abstract was available; no restriction was imposed on the language of the full-text publication. The databases were selected to provide complementary coverage of biomedical, physiological, immunological, nutritional, and interdisciplinary literature. In addition to electronic database searching, the reference lists of key primary studies and relevant reviews were manually screened, and forward citation tracking was used where appropriate to identify additional publications not retrieved through the principal database searches.

The search strategy was organized around four principal conceptual domains: (1) exercise and physical activity; (2) immunometabolism and immune regulation; (3) myokines/exerkines and mitochondrial adaptation; and (4) nutritional modulation, immune aging, inflammation, host defense, and infection. Representative search terms included “exercise,” “physical activity,” “exercise training,” “aerobic exercise,” “resistance exercise,” “interval training,” “immunometabolism,” “immune metabolism,” “immune function,” “immune regulation,” “myokine,” “exerkine,” “skeletal muscle,” “mitochondrial adaptation,” “mitochondrial biogenesis,” “mitochondrial function,” “mitophagy,” “nutrition,” “nutritional modulation,” “protein,” “amino acid,” “micronutrient,” “immune aging,” “immunosenescence,” “inflammaging,” “host defense,” “vaccination,” and “infection.” Boolean operators (AND/OR) and database-specific subject headings or controlled vocabulary were used where available. The complete database-specific search strategies, including the principal Boolean combinations, search fields, date limits, and applied search parameters, are provided in **Supplementary Table S1** to facilitate transparency and reproducibility.

The search was intentionally broad enough to capture evidence across interconnected biological levels, ranging from skeletal-muscle signaling and mitochondrial adaptation to immune-cell metabolism, nutritional regulation, immune aging, inflammation, and clinically relevant host-defense outcomes. At the same time, the search was structured around predefined conceptual domains rather than unrestricted combinations of general immunological or metabolic terms, thereby limiting retrieval of literature with only peripheral relevance to the review question. The strategy was not restricted to a single exercise modality, myokine, immune-cell population, nutritional intervention, or disease model.

Eligibility was determined according to conceptual relevance, biological level, and evidentiary contribution to the review. Eligible publications included peer-reviewed human studies, randomized and non-randomized interventions, observational studies, experimental animal studies, *in vitro*/cellular studies, and selected high-quality reviews providing essential mechanistic or conceptual context. Primary human studies were prioritized when evaluating clinical, physiological, immune, vaccine, or infection-related outcomes, whereas animal and cellular studies were used primarily to clarify mechanisms that could not be directly established in humans. Reviews were used mainly for conceptual background, terminology, and identification of relevant primary literature rather than as substitutes for original evidence. Studies were excluded when they lacked a meaningful relationship to exercise-related immunometabolic regulation, myokine/exerkine signaling, mitochondrial biology, nutritional modulation, immune aging, inflammation, or host defense, or when insufficient information was available to support interpretation.

Because the purpose of this review was integrative rather than exhaustive, study selection was guided by predefined conceptual domains and by the relevance, methodological contribution, biological level, and translational importance of the available evidence. Particular attention was given to studies that directly examined human exercise–immune interactions and to mechanistic studies capable of clarifying proposed exercise–myokine–mitochondrial–immune relationships. Recency was considered when identifying emerging concepts, but older seminal studies were retained when they provided foundational mechanistic or conceptual evidence. This approach was intended to balance contemporary evidence with established biological foundations rather than preferentially selecting recent publications.

The screening process involved title and abstract assessment followed, when appropriate, by full-text evaluation. Two authors independently screened the retrieved records and assessed potentially relevant full-text publications. Disagreements were resolved through discussion and consensus, with consultation of a third author when necessary. Based on the principal database searches and supplementary searching, approximately 5,150 records were identified across the four information sources, including 1,262 records from PubMed/MEDLINE and approximately 588 records identified through Google Scholar; the corresponding contributions from Scopus and Web of Science were approximately 1,850 and 1,450 records, respectively. After removal of duplicate records, approximately 3,900 unique records were available for title and abstract screening. Approximately 650 publications were subsequently assessed at the full-text level. The final evidence synthesis included 45 core primary studies summarized in **Tables 1**–**4**, together with additional publications used for contextual, mechanistic, and conceptual synthesis. These numbers are reported as approximate because the present review was not designed as a formal systematic review with an exhaustive, preregistered screening workflow.

**Table 1:** Exercise-regulated myokines and their immunometabolic effects on immune function and aging.

Myokine/factor	Study model and exercise stimulus	Exercise-related response	Main molecular/cellular finding	Immune relevance	Aging/host-defense relevance	Evidence level/principal limitation	Ref
IL-6	Human runners; marathon	↑ Plasma IL-6; ↑ skeletal muscle IL-6 mRNA	Exercise-associated skeletal muscle IL-6 expression; ↑ IL-1ra	Supports exercise-related cytokine–immune signaling	Potential relevance to regulation of exercise-associated inflammatory responses	Human acute exercise study; immune effects were inferred from cytokine responses rather than directly assessed through clinical immune outcomes; young/healthy runners	(29)
IL-6/TNF-α	Human marathon runners	↑ Plasma IL-6; ↑ plasma TNF-α; ↓ spontaneous monocyte cytokine production	Circulating monocytes were not the major source of post-exercise plasma IL-6/TNF-α	Supports muscle–immune cytokine crosstalk	Indicates transient modulation of systemic inflammatory signaling	Human acute exercise study; no direct assessment of long-term immune function, aging, or clinical host defense	(30)
IL-6	Healthy men; 180-min knee-extensor exercise	↑ Muscle IL-6 mRNA (~100-fold); ↑ arterial IL-6; ↑ net muscle IL-6 release	Contracting skeletal muscle released IL-6; TNF-α expression/release remained ↔	Supports IL-6 as an exercise-responsive muscle-derived immune mediator	Aging relevance not directly assessed	Human mechanistic exercise study; very small sample (n=6) and acute exercise design; no aging or clinical immune endpoint	(31)
IL-15	Healthy men; resistance exercise	↑ Muscle IL-15 mRNA (~2-fold at 24 h); ↔ muscle IL-15 protein; ↔ plasma IL-15	Exercise altered IL-15 transcription without a corresponding protein increase	Potential relevance to lymphocyte regulation; immune outcomes not directly assessed	Potential relevance to muscle–immune communication	Human acute resistance-exercise study; transcriptional response was not accompanied by protein or circulating changes; no direct immune endpoint	(32)
IL-15	Healthy men; 12-week endurance training	↑ Basal muscle IL-15 protein (~40%); ↔ muscle IL-15 mRNA; ↔ plasma IL-15	Chronic training increased intramuscular IL-15 protein without detectable circulating changes	Suggests predominantly local IL-15 adaptation	Potential relevance to long-term muscle–immune signaling	Human intervention/training study; immune outcomes and host-defense effects were not directly assessed; healthy young men	(33)
IL-15	Humans and mice; endurance exercise	↑ Exercise-associated IL-15 signaling	Muscle AMPK partly regulated IL-15; IL-15 treatment reproduced some exercise-associated tissue effects in mice	Immune effects not directly assessed	IL-15 was experimentally linked to tissue responses relevant to aging	Human + animal mechanistic evidence; aging-related effects were demonstrated mainly in skin/tissue models rather than immune aging; direct immune endpoints were not established	(34)
IL-7	Human myotubes and human strength-training participants	↑ Muscle IL-7 mRNA after 11-week training	Skeletal muscle expressed and secreted IL-7; IL-7 affected satellite-cell migration/differentiation	Potential immune relevance based on IL-7 biology; immune outcomes not directly assessed	Aging/host-defense effects not directly assessed	Human *in vitro* + intervention evidence; immune function was not directly measured; cellular findings may not translate to systemic immune effects	(35)
IL-15	Mouse model of chronic LCMV infection	↑ Muscle IL-15 during chronic infection; muscle-specific IL-15 deficiency → ↑ CD8^+^ T-cell exhaustion	Muscle-derived IL-15 supported CD8^+^CD103^+^ muscle-infiltrating lymphocytes	↑ Maintenance of functional antiviral CD8^+^ T-cell responses	↑ Antiviral immune competence; ↓ T-cell exhaustion phenotype	Animal mechanistic study; strong causal experimental evidence for muscle IL-15–T-cell interaction, but no direct exercise intervention and limited direct translation to human immune aging	(36)
IL-15	Mouse models of autoimmune myositis	↑ Muscle IL-15 following TNF-α/IFN-γ stimulation; muscle-specific IL-15 deletion → ↓ disease severity	Muscle-derived IL-15 promoted effector activity of memory-like CD8^+^ T cells	↑ CD8^+^ T-cell effector function	Relevant to immune-mediated muscle pathology rather than immune aging	Animal mechanistic study; disease-specific autoimmune model; not an exercise study and not directly representative of healthy aging or host defense	(37)
IL-6/FNDC5/METRNL	Healthy men; acute HIIE ± 20-day HIIT	Acute HIIE: ↑ IL-6 mRNA and ↑ METRNL mRNA; post-HIIT: ↑ IL-6/FNDC5 responses; ↑ resting FNDC5/METRNL	Exercise-responsive transcriptional regulation of multiple myokines	Immune effects not directly assessed	Potential relevance to systemic exercise signaling; functional immune effects remain uncertain	Human acute + short-term training study (n=9); primarily transcriptional outcomes; protein/secretion and direct immune effects were not established	(38)
Irisin/FNDC5	Humans; 26-week aerobic or strength training	↔ Circulating irisin between training groups	Training-induced increase in circulating irisin was not confirmed	Immune effects not assessed	Does not support a clear training-induced circulating irisin response	Human randomized controlled training trial; no significant between-group irisin effect; assay/storage-related measurement issues may affect interpretation; no immune endpoint	(39)
Irisin	Obese women; acute moderate/high-intensity exercise	↑ Plasma irisin during exercise	Modest exercise-associated increase; post-exercise response differed by exercise intensity	Immune effects not assessed	Aging/host-defense effects not assessed	Human randomized acute-exercise study; small sample (n=11), obese female population, and acute response only; no direct immune or aging endpoint	(40)
Irisin	Murine RAW264.7 macrophages; *in vitro*	↑ Macrophage activity/phagocytosis; ↓ respiratory-burst intensity; ↔ viability	Irisin modulated macrophage functional activity without affecting cell viability	Modulation of innate macrophage activity	Potential relevance to innate immune regulation; *in vivo*/aging effects not established	*In vitro* mechanistic evidence; no *in vivo* exercise model, human immune data, or aging population; physiological relevance of experimental irisin concentrations requires confirmation	(41)
LIF	Human males; resistance exercise and cultured human myotubes	↑ Muscle LIF mRNA (~9-fold); ↔ plasma LIF	Exercise induced local LIF expression; LIF promoted myoblast proliferation	Immune effects not directly assessed	Aging/host-defense effects not directly assessed	Human acute exercise + *in vitro* mechanistic evidence; local muscle effects demonstrated, but systemic immune and aging-related effects were not established	(42)

IL-6, interleukin-6; IL-7, interleukin-7; IL-15, interleukin-15; TNF-α, tumor necrosis factor-α; IFN-γ, interferon-γ; AMPK, AMP-activated protein kinase; LCMV, lymphocytic choriomeningitis virus; CD8^+^, cluster of differentiation 8-positive; HIIE, high-intensity interval exercise; HIIT, high-intensity interval training; FNDC5, fibronectin type III domain-containing protein 5; METRNL, meteorin-like protein; LIF, leukemia inhibitory factor; IL-1ra, interleukin-1 receptor antagonist; ROS, reactive oxygen species. ↑, increased; ↓, decreased; ↔, no significant change; —, not assessed or not applicable.5 Mitochondrial adaptation and immunometabolic remodeling.

**Table 2:** Exercise-induced mitochondrial adaptations and their impact on immune cell metabolism and function.

Exercise intervention	Mitochondrial adaptation/metabolic response	Immune-cell or functional outcome	Redox, inflammatory, or contextual outcome	Interpretation for immunometabolic remodeling	Evidence level/principal limitation	Ref
4-wk one-legged knee-extensor training + 3-h acute exercise	↑ PGC-1α transcription (10–>40-fold); ↑ PGC-1α mRNA (7–10-fold); ↑ HKII, TFAM, PPARα and citrate synthase-related metabolic signaling	Immune function not assessed	No direct redox or inflammatory endpoint assessed	Supports rapid exercise-induced transcriptional activation associated with mitochondrial adaptation in skeletal muscle	Human exercise training/mechanistic study; small sample (n=7); skeletal-muscle mitochondrial signaling was assessed without direct immune outcomes	(43)
Acute low-volume high-intensity interval exercise	↑ p38 MAPK/AMPK activation; ↑ nuclear PGC-1α at 3 h; ↑ mitochondrial gene expression; ↑ mitochondrial protein content and enzyme activity at 24 h	Immune function not assessed	Redox/immune effects not directly assessed	Supports a temporal sequence linking exercise signaling to mitochondrial biogenesis	Human acute exercise/mechanistic study; small sample (n=8); no direct immune or inflammatory endpoint	(44)
Acute exercise	↑ Nuclear translocation/activation of PGC-1α; ↑ NRF-1/NRF-2 promoter binding; ↑ mitochondrial gene transcripts before ↑ PGC-1α protein	Immune function not assessed	Redox/immune effects not assessed	Suggests that activation of existing PGC-1α may precede later increases in mitochondrial content	Human acute exercise/mechanistic study; primarily molecular/transcriptional evidence; no direct immune or aging endpoint	(45)
2-h endurance cycling at 70% VO_2_peak; fed vs fasted	↑ PGC-1α/TFAM/HSP60-related mitochondrial biogenesis markers; ↑ fission in fed state; ↔ major mitophagy markers during early recovery	Immune function not assessed	Fission increased selectively in the fed state; major mitophagy markers were largely unchanged during early recovery	Indicates that nutritional state modifies mitochondrial remodeling and that mitochondrial biogenesis does not necessarily coincide with acute mitophagy activation	Human acute endurance-exercise study in trained athletes (n=7); early post-exercise sampling limits conclusions about later mitophagy responses	(46)
6-wk HIIT or MICT	↑ Mitochondrial membrane potential; ↑ maximal and reserve O_2_ consumption; ↑ bioenergetic health index; ↓ mitochondrial matrix oxidant burden	↑ Perforin and granzyme B; altered exercise-induced NK-cell mobilization	↓ Exercise-associated mitochondrial oxidant burden	Provides direct human evidence linking exercise training with improved NK-cell mitochondrial bioenergetics and cytotoxic phenotype	Human randomized controlled exercise trial (n=60); young healthy sedentary men; immune and mitochondrial outcomes were assessed, but clinical host-defense outcomes were not	(47)
6-wk HIIT or MICT + hypoxic exercise challenge	↑ Mitochondrial OCR; ↑ citrate synthase and succinate dehydrogenase activity; ↑ mitochondrial membrane potential; ↑ mitofusin/DRP-1 ratio; ↓ matrix oxidant burden	↓ Circulating senescent lymphocytes following hypoxic exercise	↓ Mitochondrial oxidative burden under hypoxic stress	Suggests that exercise training may improve lymphocyte mitochondrial resilience under metabolic/hypoxic challenge	Human randomized controlled exercise trial (n=60); young sedentary men; effects were assessed under an experimental hypoxic challenge rather than in clinical populations	(48)
Acute maximal exercise	↑ Routine cellular O_2_ flux; ↔ LEAK, OXPHOS and ET capacities at cellular level; ↑ tissue-level O_2_ flux after accounting for PBMC mobilization	↑ Mobilization of central-memory and senescent CD8^+^ T cells	Immune-cell redistribution substantially influenced tissue-level bioenergetic changes	Indicates that acute exercise-associated changes in whole-PBMC bioenergetics may partly reflect altered immune-cell composition rather than intrinsic mitochondrial adaptation	Human acute exercise/mechanistic study (n=11); collegiate swimmers; acute design and PBMC heterogeneity limit inference about intrinsic cell-specific mitochondrial adaptation	(49)
Chronic moderate-intensity exercise training	↑ Mitochondrial quality; ↑ reliance on oxidative phosphorylation; ↓ mitochondrial ROS production	↓ LPS-induced NF-κB activation and pro-inflammatory gene expression; ↑ M2-like-associated genes	↓ Mitochondrial ROS; attenuated inflammatory response	Supports exercise-associated metabolic and epigenetic remodeling of macrophages consistent with a trained-immunity phenotype	Animal exercise/mechanistic study; mouse model with *ex vivo* macrophage analysis; translation to human immune aging remains uncertain	(50)
4-wk voluntary exercise in mice; SPM treatment in murine/human macrophages	↑ Macrophage mitochondrial respiration following exercise; RvD1/RvE1/MaR1 → ↑ mitochondrial respiration via AMPK	Potential shift toward pro-resolving macrophage metabolism	↑ Proresolving lipid mediators; ↓ inflammatory lipid mediator balance	Suggests an exercise-associated lipid mediator–AMPK pathway linking systemic exercise signals to macrophage mitochondrial metabolism	Animal exercise + *in vitro* mechanistic evidence; causal pathway supported experimentally, but direct human exercise–immune translation remains unestablished	(51)

AMPK, AMP-activated protein kinase; PGC-1α, peroxisome proliferator-activated receptor γ coactivator-1α; NRF-1/2, nuclear respiratory factors 1/2; TFAM, mitochondrial transcription factor A; HKII, hexokinase II; HSP60, heat shock protein 60; HIIT, high-intensity interval training; MICT, moderate-intensity continuous training; NK, natural killer; OCR, oxygen consumption rate; OXPHOS, oxidative phosphorylation; ET, electron transfer; PBMC, peripheral blood mononuclear cell; ROS, reactive oxygen species; NF-κB, nuclear factor κB; LPS, lipopolysaccharide; SPMs, specialized proresolving mediators; RvD1, resolvin D1; RvE1, resolvin E1; MaR1, maresin 1. ↑, increased; ↓, decreased; ↔, no significant change.

**Table 3:** Nutritional modulators of exercise-induced immunometabolic reprogramming and immune aging.

Population/design	Nutritional intervention	Exercise/training protocol	Immune/metabolic outcomes	Redox, inflammatory, or contextual outcome	Main findings	Evidence level/principal limitation	Ref
32 trained male triathletes; randomized dietary groups; 6 d	High-CHO diet (~12 g/kg/day) vs self-selected diet	1 h running at 70% VO_2_max daily for 6 d during increased workload	Salivary IgA; blood glucose	Cortisol	High-CHO: cortisol response to exercise ↓; post-exercise s-IgA ↑; blood glucose maintained ↔. Findings suggest attenuation of selected stress/immune changes, without direct evidence of reduced infection risk.	Human randomized exercise–nutrition intervention; trained male endurance athletes; short duration and surrogate immune endpoints; no direct infection outcome.	(52)
11 elite triathletes; randomized repeated-measures crossover	Periodized CHO/”sleep-low” vs evenly distributed CHO	4-d cycling–running training blocks	s-IgA; IL-6	Hepcidin	Exercise-induced IL-6 ↑, but no diet effect; hepcidin ↑ post-exercise, with a larger response after LOW-CHO exposure on day 2; s-IgA secretion ↔/↑ depending on trial day. Overall, short-term low CHO availability produced limited changes in measured immune markers.	Human randomized crossover intervention; very small sample and short exposure; trained triathletes; mainly acute biomarker outcomes.	(53)
10 male athletes; randomized placebo-controlled crossover	Oral glutamine vs placebo	2 h cycling at 75% VO_2_max	Lymphocyte trafficking; NK/LAK activity; T-cell proliferation; plasma glutamine	Neutrocytosis	Glutamine supplementation prevented the exercise-associated fall in plasma glutamine ↔/maintained, but lymphocyte trafficking and functional immune outcomes were largely ↔; neutrocytosis was modestly ↓. No convincing evidence for a major immunoprotective effect.	Human randomized placebo-controlled crossover mechanistic study; small sample; acute exercise model; no clinical immune or infection endpoint.	(54)
10 resistance-trained male collegiate athletes; randomized double-blind crossover	CHO 1 g/kg vs placebo	Acute resistance exercise	s-IgA; IL-2; IL-5	–	s-IgA ↔; IL-2 ↔; IL-5 ↓ after exercise, with no meaningful CHO × time effect. CHO ingestion did not materially alter the measured salivary immune response.	Human randomized crossover intervention; small, young male athletic sample; single acute exercise bout; limited immune endpoints.	(55)
27 recreationally active men/women; randomized controlled trial	Hydrolyzed beef protein vs whey protein vs isoenergetic CHO, 8 wk	Resistance training	Salivary HNP1–3	Blood health indices; training outcomes	HNP1–3 concentration/secretion ↓ in the beef-protein group from baseline; no consistent between-group immune differences. Protein supplementation did not demonstrate a clear generalized immune benefit.	Human randomized controlled training–nutrition trial; small sample; salivary immune marker; no direct immune-function or clinical endpoint.	(56)
20 male athletic students; randomized double-blind	Vitamin E 450 mg/day for 8 wk vs placebo	Cycling to exhaustion	–	MDA; protein carbonyls; CK; endurance performance	MDA ↔; protein carbonyls ↔; CK ↔; cycling performance ↔. Vitamin E did not significantly modify measured oxidative-stress or muscle-damage outcomes.	Human randomized placebo-controlled intervention; small male sample; high-dose vitamin E; oxidative-stress and muscle-damage surrogates rather than immune outcomes.	(57)
64 trained female athletes; randomized double-blind trial	Vitamin C, vitamin E, combined C+E, or placebo for 4 wk	Strenuous aerobic exercise/training	–	CK; AST; LDH; MDA	Oxidative-stress markers ↓ in vitamin C, C+E and control groups; CK ↓ in vitamin C group; LDH ↓ in vitamin E group. Effects were outcome- and treatment-specific, limiting broad conclusions regarding antioxidant protection.	Human randomized clinical trial; trained female athletes; short intervention; predominantly biochemical muscle-damage/oxidative-stress endpoints; no direct immune endpoint.	(58)
455 healthy men/women; randomized vitamin E vs placebo	Vitamin E 800 IU during 8-wk aerobic program	Aerobic/cardiovascular exercise training	Inflammatory markers	Lipids; oxidative-stress markers; VO_2_	No significant vitamin E-related improvement in oxidative-stress or inflammatory markers; exercise-associated favorable trends occurred in both groups. CRP ↓ correlated with VO_2_ ↑. Results do not support a clear additional benefit from high-dose vitamin E.	Large human randomized exercise–nutrition trial; relatively broad healthy population; high-dose vitamin E; no direct immune-cell or clinical host-defense endpoint.	(59)
38 physically trained adults; double-blind placebo-controlled trial	DHA 715 mg/day for 12 wk	Eccentric cycling-induced muscle damage	IL-6; TNF-α; IL-10	Omega-3 Index; DOMS; neuromuscular function	Omega-3 Index ↑; DOMS ↓ at 24–48 h; selected inflammatory cytokine AUCs showed interaction effects (IL-6, TNF-α, IL-10), but these findings do not establish a uniform anti-inflammatory effect.	Human randomized placebo-controlled intervention; trained adults; acute post-exercise inflammatory model; cytokine changes do not by themselves establish broader immune protection.	(60)
26 recreationally active adults; randomized double-blind controlled trial	Polyphenol-rich foods vs lower-polyphenol foods for 3 d + pre-exercise dose	Strenuous muscle-damaging exercise	IL-6; IL-10; TNF-α; other cytokines	GPX; 8-OHdG; plasma polyphenols	Plasma polyphenols ↑ markedly; inflammatory responses were mixed/no consistent difference; urinary 8-OHdG ↑ with high-polyphenol intervention, whereas erythrocyte GPX activity was ↓. Overall effects on acute inflammation and oxidative stress were limited and inconsistent.	Human randomized placebo-controlled exercise–nutrition trial; small recreationally active sample; short dietary exposure; outcomes limited to acute inflammatory and oxidative-stress biomarkers.	(61)

CHO, carbohydrate; s-IgA, salivary immunoglobulin A; IL, interleukin; NK, natural killer; LAK, lymphokine-activated killer; HNP1–3, human neutrophil peptides 1–3; MDA, malondialdehyde; CK, creatine kinase; LDH, lactate dehydrogenase; AST, aspartate aminotransferase; DHA, docosahexaenoic acid; GPX, glutathione peroxidase; 8-OHdG, 8-hydroxy-2′-deoxyguanosine; VO_2_max, maximal oxygen uptake; DOMS, delayed-onset muscle soreness; CRP, C-reactive protein; AUC, area under the curve. ↑, increased; ↓, decreased; ↔, no significant change; —, not assessed or not applicable.

**Table 4:** Exercise-induced immunometabolic remodeling, immune aging, and host defense across immune cell populations.

Population	Exercise protocol	Immune outcome	Redox, inflammatory, or contextual outcome	Main finding	Interpretation	Evidence level/principal limitation	Ref
Older adults ≥64 y	Moderate aerobic exercise, 65–75% HRR, 25–30 min, 3×/wk, 10 mo	Influenza antibody; Granzyme B	–	Greater antibody fold-increase and higher Granzyme B activity in exercise group	Potential enhancement of vaccine-related humoral and cytotoxic responses	Human randomized controlled exercise intervention; small sample (n=27); older adults; vaccine-response and cytotoxic biomarkers rather than clinical protection.	(62)
Healthy adults	15-min arm/shoulder exercise immediately before pneumococcal vaccination	Antibody responses to 11 pneumococcal strains	–	Exercise enhanced antibody responses, particularly after half-dose vaccination	Adjuvant effect may be more evident when baseline antigenic stimulation is weaker	Human randomized controlled vaccine–exercise trial; young healthy population and acute intervention; findings may depend on baseline antigenic stimulation and may not generalize to older or high-risk populations.	(63)
Physically active/inactive older adults	Acute cardiorespiratory vs resistance exercise	T-cell subsets, differentiated CD8+, Th17	–	Exercise mobilized multiple T-cell subsets; responses differed by exercise modality and training status	Acute immune-cell redistribution is dependent on exercise mode and training status	Human randomized crossover mechanistic study; small sample (n=24); acute redistribution rather than long-term immune function or clinical host defense.	(64)
Adults; influenza/COVID-19 vaccination models	90-min light–moderate aerobic exercise after vaccination	Vaccine-specific serum antibody	Experimental IFN-α contribution	Higher antibody responses after exercise; IFN-α implicated experimentally	Exercise may act as a behavioral vaccine adjuvant, although mechanistic contribution remains uncertain	Human exercise–vaccination experiments plus animal mechanistic evidence; heterogeneous vaccine models and exercise protocols; mechanistic contribution of IFN-α in humans remains uncertain.	(65)
Adults >65 y	12-week light–moderate exercise	COVID-19 vaccine antibody	–	Exercise group showed higher antibody concentrations at 15 days and 12 weeks	Suggestive of improved antibody response, but small sample limits inference	Human randomized controlled intervention; small sample (n=30); antibody concentration alone does not establish improved clinical vaccine effectiveness.	(66)
Older CMV-seropositive adults	≥12 mo combined exercise training	Influenza-specific antibodies; CMV IgG; CD8+ T cells	IL-6; IL-10; IL-10/IL-6 ratio	Exercise-trained participants showed altered CD8+ T-cell profiles and higher IL-10/IL-6 ratio; vaccine-response associations varied by CMV status	Exercise-associated immune remodeling may interact with CMV burden and inflammatory context	Human observational exercise-status comparison; non-randomized design and potential residual confounding; CMV burden may modify immune responses.	(67)
Older resistance-training-naïve adults	Acute eccentric resistance exercise before influenza vaccination	Antibody titers; influenza-specific T cells	–	No significant enhancement of antibody or T-cell responses	Acute exercise does not consistently enhance vaccine immunity	Human randomized controlled trial; small sample (n=29); single acute exercise bout; results may not generalize to other exercise doses or training states.	(68)
Young untrained men	30 min at 35–75% VO_2_max; 30–90 min at 55% VO_2_max	Salivary IgA	Salivary cortisol	Higher intensity and longer duration reduced s-IgA secretion acutely	Mucosal immune responses may depend on exercise dose	Human randomized crossover acute exercise study; very small sample (n=12); young untrained men; acute salivary endpoint with uncertain implications for infection risk.	(69)
Elite cross-country skiers	18-day living-high/training-low protocol	Salivary sIgA	Salivary protein; cortisol	sIgA decreased during hypoxic training and remained lower after recovery	Combined exercise and hypoxic stress may transiently impair mucosal immunity	Human controlled training/hypoxia study; very small sample (n=11); elite athletes; combined exercise and hypoxic exposure makes independent effects difficult to separate.	(70)
Adults ≥50 y	Exercise training vs MBSR vs control	Influenza antibody, nasal IgA, IFN-γ, IL-10	Psychosocial factors	Exercise did not significantly improve vaccine responses	Immune benefits are not universal and may depend on host and intervention characteristics	Human randomized controlled intervention; relatively heterogeneous older population; no significant exercise effect on vaccine outcomes; psychosocial and host factors may contribute to response variability.	(71)
Healthy young adults	Acute eccentric exercise immediately, 6 h, or 48 h before influenza vaccination	Antibody and IFN-γ responses	–	No enhancement of antibody or cell-mediated responses	Timing and baseline vaccine responsiveness may constrain the adjuvant effect	Human randomized controlled trial; large sample (n=156) but young healthy population; high baseline vaccine responsiveness may constrain detection of an additional exercise effect.	(72)
Older adults	Aerobic exercise for 10 mo	Influenza antibody and IFN-γ	Psychosocial factors	Exercise increased antibody and IFN-γ responses; psychosocial factors partly explained IFN-γ changes	Exercise-related immune enhancement may involve both physiological and psychosocial pathways	Human randomized controlled exercise intervention; small sample; psychosocial factors and physiological mechanisms are difficult to fully disentangle; immune biomarkers do not directly establish clinical protection.	(73)

HRR, heart rate reserve; IgA, immunoglobulin A; IgG, immunoglobulin G; IgM, immunoglobulin M; sIgA, secretory immunoglobulin A; CMV, cytomegalovirus; IL, interleukin; IFN-γ, interferon-gamma; IFN-α, interferon-alpha; CD, cluster of differentiation; Th17, T helper 17; HAI, hemagglutination inhibition; MFI, mean fold increase; VO_2_max, maximal oxygen uptake; MBSR, mindfulness-based stress reduction; —, not assessed or not applicable. ↑, increased; ↓, decreased; ↔, no significant change.

No formal systematic-review risk-of-bias instrument, such as RoB 2 or SYRCLE, was applied across the heterogeneous evidence base because the present work was designed as a structured narrative review rather than a systematic review with quantitative synthesis. Nevertheless, methodological quality, study limitations, and the strength and directness of the available evidence were considered during interpretation. Evidence was categorized according to biological and clinical level, including (i) direct human clinical or physiological evidence, (ii) human associative or observational evidence, (iii) human intervention evidence without direct mechanistic confirmation, (iv) animal or other preclinical mechanistic evidence, (v) cellular/*in vitro* evidence, and (vi) integrative or hypothesized mechanistic links. This evidence-level framework was used to avoid treating preclinical mechanisms or associative biomarker findings as equivalent to demonstrated clinical effects.

The literature was also interpreted with attention to important sources of heterogeneity, including acute versus chronic exercise; aerobic/endurance, resistance, interval, or combined training; exercise intensity, duration, and volume; training status; age; sex and hormonal status; nutritional and energy-availability state; metabolic health; habitual physical activity; and the presence of obesity, diabetes, or other relevant comorbidities. Where applicable, potential confounding factors such as medication use, smoking, sleep, psychological stress, circadian timing, recent infection or vaccination, habitual activity, protein and micronutrient intake, and other metabolic or lifestyle characteristics were considered when interpreting human observational and intervention evidence. Reverse causation, healthy-participant bias, baseline differences, and differences in training status were also considered where relevant.

Data and concepts extracted or qualitatively considered from the selected literature included study population or experimental model, age and sex characteristics, exercise modality and dose, intervention duration, nutritional context, myokines/exerkines and signaling mediators, mitochondrial adaptations, immune-cell populations, metabolic pathways, inflammatory outcomes, markers of immunosenescence or inflammaging, vaccine-related responses, infection-related outcomes, and other measures relevant to host defense. Particular attention was given to whether an outcome represented a molecular biomarker, a cellular functional measure, a physiological immune response, a vaccine-related endpoint, or a clinically documented infection-related outcome. Accordingly, changes in circulating cytokines, immune-cell phenotypes, mitochondrial respiration, metabolic markers, or other surrogate measures were not assumed to indicate improved immune competence or clinical protection in the absence of corresponding functional, vaccine-related, infection-related, or clinical evidence.

The synthesis was thematic, comparative, and evidence-stratified rather than quantitatively pooled. Evidence was organized around the principal conceptual relationships among exercise, skeletal-muscle signaling, mitochondrial adaptation, nutritional context, immune-cell metabolism, immune aging, inflammation, and host defense. Direct human findings were distinguished from associative evidence and from mechanistic extrapolations derived from animal or cellular models. In particular, evidence for an exercise-induced change in skeletal-muscle mitochondrial function or circulating myokines was not interpreted as direct evidence of equivalent remodeling in immune cells unless this was experimentally demonstrated. Similarly, changes in inflammatory biomarkers were not interpreted as evidence of improved antimicrobial or antiviral protection without appropriate functional, vaccine-related, infection-related, or clinical endpoints. Where multiple studies addressed related biological pathways, findings were synthesized according to the consistency of the direction of evidence, biological plausibility, evidence level, and translational relevance rather than by assigning a quantitative summary effect.

Finally, the limitations inherent to the narrative-review design were explicitly considered. The review does not constitute an exhaustive systematic review, does not provide pooled effect estimates, and does not replace formal study-level risk-of-bias assessment. The heterogeneous nature of the evidence, including differences in exercise protocols, populations, nutritional states, biological models, outcome measures, and myokine or mitochondrial assays, limits direct comparison across studies. In addition, the use of multiple databases with substantial overlap, the approximate nature of the screening counts, and the selective emphasis on studies providing mechanistically or clinically informative evidence may introduce selection and interpretive bias. These methodological constraints were incorporated into the interpretation of the evidence and the formulation of translational implications.

## 3 Exercise-driven immunometabolic reprogramming: an integrative framework

Exercise can be viewed as a recurring physiological disruption that, for a limited time, shifts the availability, use, and distribution of metabolic resources across multiple tissues. From an immunometabolic standpoint, these shifts may act as cues that shape how immune cells respond to changes in energy demand and the availability of substrates. Accordingly, rather than reflecting a single pathway, exercise-associated immunometabolic reprogramming can be interpreted as a coordinated reconfiguration encompassing cellular metabolism, systemic signaling, and inter-tissue communication (4, 74, 75). This interpretation is consistent with the emerging field of exercise immunometabolism, in which exercise is considered capable of modifying both systemic metabolic signals and the metabolic plasticity of immune cells (22, 76). A key element of this framework is metabolic flexibility. During physical activity, rising energy needs coincide with time-varying changes in substrate utilization, circulating metabolites, hormonal signals, and mediators released from tissues. Such alterations can reshape the metabolic milieu in which immune cells function. Because immune-cell activity is tightly linked to metabolic status, changes in nutrient supply and in cellular energy pathways can affect processes including proliferation, differentiation, migration, and effector function (6, 77). However, the magnitude and direction of these responses are not uniform across immune-cell subsets or exercise conditions, and changes observed in circulating immune cells should not automatically be interpreted as equivalent changes in tissue-resident immune populations (22). Thus, the immune response to exercise may reflect not only direct signaling events but also adaptation to a changing metabolic environment.

Importantly, immunometabolic reprogramming should not be treated as equivalent to immune activation or immune suppression. Acute exercise and repeated exercise training involve biologically distinct scenarios. One session of exercise can trigger short-lived metabolic shifts and cellular redistribution, while repeated exposure may foster adaptation to recurrent physiological stress (9, 25). Human and translational evidence indicates that acute exercise can rapidly alter immune-cell trafficking and metabolic signaling, whereas repeated exercise may produce longer-term adaptations; these responses depend substantially on exercise modality, intensity, duration, recovery, training status, and age (9, 18, 22). The overall response is therefore likely to vary with exercise modality, intensity, duration, recovery, baseline fitness, and the individual’s physiological status. Aerobic/endurance, resistance, interval, and combined exercise should therefore not be assumed to generate interchangeable immunometabolic responses, and findings from young, trained, or athletic populations should not automatically be extrapolated to older or sedentary adults (18, 76). Recognizing this difference may help account for why immune findings that appear inconsistent have been reported across different exercise studies.

The framework also highlights communication across tissues rather than treating immune cells as separate entities. Skeletal muscle, liver, adipose tissue, and immune compartments continuously exchange metabolites and signaling molecules, forming a dynamic network in which local metabolic shifts can affect systemic physiology (74, 78). Although skeletal muscle is an important source of exercise-responsive signaling molecules, circulating exerkines are not exclusively muscle-derived; exercise-responsive mediators may also originate from or be influenced by adipose tissue, liver, bone, vascular tissues, and other organs (1, 2). Accordingly, an increase in a circulating mediator after exercise does not by itself establish skeletal-muscle origin, and muscle expression, secretion, circulating concentration, receptor engagement, and demonstrated effects on immune-cell function should be regarded as distinct levels of evidence (2). Exercise can reshape this network by changing the timing and strength of these signals, which may influence the metabolic environment in which immune responses take place.

Mitochondrial adaptation should also be evaluated across multiple levels of biological organization. Exercise-related alterations in mitochondrial signaling, biogenesis-linked pathways, respiratory capacity, redox balance, dynamics, or mitophagy may not represent equivalent end points, and findings of adaptation in skeletal muscle do not automatically imply comparable remodeling in immune cells. Therefore, higher expression of a mitochondrial regulatory protein or a biogenesis-associated marker should not be taken as conclusive evidence for increased mitochondrial content, completed mitochondrial turnover, or improved mitochondrial function unless appropriate functional assessments are provided. This distinction is especially important for the present framework because much of the mechanistic evidence connecting exercise to mitochondrial adaptation comes from skeletal muscle or experimental models, whereas direct demonstrations of coordinated mitochondrial remodeling in human immune-cell populations are still relatively scarce (22, 76).

Taken as a whole, exercise-induced immunometabolic reprogramming is best understood as a multiscale, context-dependent framework rather than as a single, well-established signaling pathway. It links acute physiological stress and chronic training adaptations to alterations in substrate availability, signaling between organs, metabolic flexibility of immune cells, and, possibly, longer-term aspects of immune aging. Crucially, the framework distinguishes directly evidenced human responses from associative observations and from mechanistic propositions drawn from animal or cellular studies. This separation supports the assessment of particular myokines, mitochondrial mechanisms, nutritional modulators, and immune-cell pathways in the sections that follow, without presuming that an adaptation detected in one tissue will necessarily yield a comparable functional outcome in another (7, 79).

## 4 Skeletal muscle–immune crosstalk: myokines as exercise-induced immune mediators

Skeletal muscle is increasingly understood as an active participant in communication between organs. Exercise modifies the expression and secretion of multiple cytokines and peptide mediators that could affect the regulation of the immune system. However, the current body of evidence does not support a single, consistent model in which exercise merely elevates circulating myokines and thereby improves immune function. Rather, the results suggest a communication framework that depends on context, in which the size of the myokine response, its timing, the tissue compartment involved, and the biological form of the myokine may be more informative than alterations in circulating concentrations alone (**Table 1**). Importantly, the term “myokine” should be applied cautiously because some exercise-responsive mediators are produced by multiple tissues, and circulating concentrations do not necessarily establish skeletal-muscle origin or biological activity at a target tissue (2). Moreover, muscle gene expression, intracellular protein abundance, secretion, circulating concentration, receptor engagement, and a demonstrable functional effect in immune cells represent distinct levels of evidence and should not be treated as equivalent.

### 4.1 Exercise-induced myokine signaling

Among exercise-responsive myokines, IL-6 offers well-established evidence for communication between skeletal muscle and the immune system. Sustained running and isolated knee-extensor exercise have been linked to pronounced increases in skeletal muscle IL-6 expression and to net release from contracting muscle, whereas TNF-α did not display a similar pattern (29–31). Direct arteriovenous measurements across exercising limbs provide particularly strong evidence that contracting skeletal muscle can contribute to circulating IL-6 during prolonged exercise (80, 81). These findings align with the idea that contracting muscle can directly shape the cytokine milieu during exercise, rather than merely reacting to inflammatory cues produced elsewhere. At the same time, the size of the IL-6 response changes markedly depending on exercise duration, intensity, the amount of muscle mass recruited, and metabolic stress, which constrains the ability to view IL-6 as a consistently beneficial or harmful mediator. Indeed, intramuscular glycogen availability can further modify exercise-induced IL-6 expression and release, illustrating that the response is influenced by metabolic context as well as exercise dose (81).

IL-15 highlights an additional level of complexity. Resistance exercise raised skeletal muscle IL-15 mRNA without any parallel increase in muscle protein or circulating IL-15, while extended endurance training was linked to higher basal muscle IL-15 protein in the absence of detectable changes in plasma concentrations (32, 33). Together, these results indicate that regulation of exercise-related myokines may occur mainly within the muscle compartment, and that transcriptional responses should not necessarily be taken to reflect enhanced systemic signaling. This distinction is particularly important for IL-15 because human studies have reported discordant responses at the mRNA, muscle-protein, and circulating-protein levels (33). Compartment-specific considerations similarly apply to IL-7, for which skeletal muscle expression and secretion have been shown, and muscle IL-7 mRNA increased after strength training (35). Nevertheless, these studies did not determine direct immune effects. Thus, evidence for exercise-induced regulation of a myokine within skeletal muscle should not be interpreted as evidence of a corresponding change in immune-cell exposure or function.

Evidence for irisin is especially heterogeneous. Acute exercise has been linked to small increases in circulating irisin, while a randomized training study found no significant, training-related rise in circulating irisin (39, 40). Regulation of FNDC5 and METRNL transcripts in response to exercise has also been reported after high-intensity interval exercise and short-term training (38). Taken together, these results imply that exercise responsiveness may vary depending on whether the outcome is measured at the mRNA level, the intracellular protein level, or the circulating level. They further suggest that the biological relevance of alterations in specific myokines should not be concluded solely from their designation as exercise-responsive factors. Interpretation of circulating irisin requires additional methodological caution because differences among immunoassays, antibody specificity, and analytical approaches have contributed to inconsistencies in the human literature (82). Mass-spectrometry-based measurements have provided evidence that circulating human irisin can be detected and may respond to exercise, but methodological differences remain relevant when comparing studies (83).

### 4.2 Myokines and innate immunity

The possible link between myokines and innate immunity is backed by experimental findings, yet it is still less firmly supported than the exercise-driven control of myokine expression itself. In macrophage cultures, irisin enhanced phagocytic activity while lowering the intensity of the respiratory burst, with no observable impact on cell viability (41). This profile is consistent with shifts in macrophage functional state; however, the *in vitro* nature of the study limits any direct inference regarding systemic immune responses after exercise. Notably, the existing exercise studies on irisin did not directly demonstrate that exercise-related changes in circulating irisin lead to corresponding alterations in macrophage function (38–41). The distinction is therefore between an experimentally demonstrated effect of exogenous irisin on cultured macrophages and an unproven causal chain linking exercise, muscle-derived irisin secretion, circulating exposure, and macrophage function *in vivo*.

IL-6 offers another example of the difference between exercise-triggered cytokine release and subsequent immune consequences. After prolonged running, circulating monocytes were not detected as the principal source of the post-exercise rise in plasma IL-6 and TNF-α, and monocyte cytokine responsiveness to lipopolysaccharide was modified (30). This result is consistent with bidirectional communication between systemic signals associated with exercise and immune cells, but it does not demonstrate that muscle-derived IL-6 by itself explains the immune changes that were observed. Accordingly, the relationship is better understood as a coordinated response that involves muscle activity, endocrine stress signals, and redistribution of immune cells. In addition, IL-6 is not exclusively a muscle-derived mediator; adipose tissue and immune cells can also contribute to circulating IL-6, further limiting attribution of systemic immune effects to skeletal-muscle IL-6 alone (2).

### 4.3 Myokines and adaptive immunity

The more direct preclinical mechanistic evidence currently connecting signaling derived from skeletal muscle to adaptive immunity involves IL-15. Experimental work suggests that IL-15 produced by muscle can modulate CD8^+^ T-cell biology. In chronic lymphocytic choriomeningitis virus infection, loss of IL-15 specifically in muscle correlated with increased CD8^+^ T-cell exhaustion, while IL-15 originating from muscle supported a subset of CD8^+^CD103^+^ muscle-infiltrating lymphocytes displaying characteristics consistent with retained proliferative capacity (36). Together, these results indicate that skeletal muscle can contribute to local control of antiviral T-cell responses. However, the infection model and genetic approaches used in this study do not demonstrate that exercise-induced IL-15 elicits the same effect in humans. Accordingly, this study provides evidence for a muscle-derived IL-15–T-cell mechanism in a specific murine infection model, rather than direct evidence that exercise-induced IL-15 improves antiviral immunity in humans (36).

Additional experimental evidence suggests that IL-15 produced by inflamed skeletal muscle can enhance effector activity in memory-like CD8^+^ T cells and thereby contribute to autoimmune myositis in mice (37). Taken together with human studies demonstrating exercise-associated changes in intramuscular IL-15 (32, 33), these findings support a credible biological link between exercise-responsive muscle signaling and lymphocyte regulation. However, the direction of this pathway and its functional outcomes seem to be highly dependent on tissue context. IL-15 signaling might promote antiviral immune competence in one context while driving pathological immune activation in another. This dependence on context argues against describing IL-15 as inherently “immunoprotective.” Importantly, the human exercise studies establish changes in muscle IL-15 expression or protein abundance, whereas the immune-functional effects described in (36, 37) arise primarily from experimental infection or inflammatory models. Thus, the proposed exercise–IL-15–T-cell pathway should currently be regarded as biologically plausible but not clinically established.

### 4.4 Myokines, inflammation, and immune aging

The link between myokines and immune aging is best viewed as an area still developing rather than a fully settled field. Experimentally, exercise-induced IL-15 has been shown to be involved in signaling between skeletal muscle and aging tissues, where AMPK-dependent muscle signaling supports the regulation of IL-15 and administration of IL-15 reproduces some exercise-associated effects in aging skin models (34). Taken together, these results indicate that signals originating from muscle may contribute to systemic adaptations elicited by exercise; however, they provide no evidence that IL-15 reverses immune aging or restores immune competence in older humans. Indeed, the IL-15 studies underlying these observations primarily examined skin metabolism, mitochondrial adaptation, or experimental aging models rather than validated measures of human immunosenescence or clinically meaningful host defense (34).

A wider reading of the available evidence suggests that exercise can change the inflammatory milieu via repeated, short-lived metabolic and cytokine signals, rather than through a sustained increase in any single myokine. Accordingly, it is essential to distinguish between immediate exercise responses and long-term training adaptations. Acute exercise may trigger marked alterations in cytokine expression or circulating levels, while repeated training can adjust baseline tissue expression without generating comparable systemic changes (32, 33, 38). Recent systematic evidence further indicates substantial heterogeneity in exercise-induced myokine responses according to the specific myokine, exercise mode, sampling time, and study design, with many reported changes being modest or transient (84). Additional factors including age, sex, fitness level, exercise modality, nutritional status, disease status, and methodological differences in myokine assessment, may also explain the variability observed across studies. These factors are particularly relevant when extrapolating findings from young or physically trained participants to older adults, because age-related changes in myokine regulation and immune-cell composition may modify the relationship between exercise and immune signaling (76).

Several translational constraints should therefore be recognized. Evidence from human studies is most compelling for exercise-related changes in muscle measurements or circulating myokine levels, while direct evidence connecting these changes to clinically significant shifts in immune aging is still comparatively scarce. Moreover, many mechanistic studies rely on cell culture systems or animal models, which offer valuable causal insights but cannot automatically be extended to human exercise physiology (36, 37, 41). In addition, circulating myokine concentrations should be interpreted as molecular or systemic biomarkers unless accompanied by evidence of receptor engagement, target-cell signaling, functional immune responses, or clinically relevant host-defense outcomes. Changes in circulating IL-6, IL-15, irisin, or related mediators therefore should not, by themselves, be interpreted as evidence of improved antimicrobial or antiviral protection. Future work that integrates longitudinal exercise interventions with validated assessments of myokine secretion, immune-cell phenotype, metabolic status, and functional host-defense outcomes will be required to establish whether exercise-induced muscle signaling constitutes a clinically meaningful mechanism of immune adaptation. Such studies should also distinguish acute from chronic responses and incorporate sex, age, exercise modality, nutritional status, and relevant clinical or metabolic context when evaluating these pathways.

Mitochondrial adaptation offers an important mechanistic connection between repeated exposure to exercise and changes in cellular metabolic capacity. However, the current body of evidence does not substantiate a straightforward model in which exercise uniformly enhances mitochondrial function across all tissues and immune-cell populations. Instead, exercise seems to activate a temporally sequenced and context-dependent remodeling program that includes mitochondrial biogenesis, organelle dynamics, oxidative phosphorylation, redox regulation, and, possibly, mitochondrial quality control (**Table 2**). Notably, much of the mechanistic evidence has been produced in skeletal muscle, while direct evidence from immune cells remains relatively limited. This difference matters when evaluating how exercise-associated skeletal muscle adaptation may relate to immune function. Importantly, mitochondrial biogenesis, respiratory capacity, mitochondrial dynamics, and mitophagy represent related but non-equivalent processes; changes in one of these domains should not automatically be interpreted as evidence of improved mitochondrial quality or completed mitochondrial turnover. Similarly, increased mitochondrial capacity in skeletal muscle does not establish comparable remodeling in leukocytes or other immune-cell populations.

### 5.1 Exercise-induced mitochondrial biogenesis and quality control

In human skeletal muscle, acute exercise rapidly triggers signaling pathways linked to mitochondrial remodeling. The exercise-driven activation and nuclear redistribution of PGC-1α, along with heightened activity of upstream kinases such as AMPK and p38 MAPK, occur before multiple downstream transcriptional and enzymatic alterations associated with mitochondrial function (43–45). For example, acute endurance exercise in trained men increased nuclear PGC-1α abundance together with activation of AMPK- and p38 MAPK-related signaling, whereas whole-muscle PGC-1α abundance did not increase immediately (86). Similarly, brief high-intensity interval exercise activated AMPK and p38 MAPK, followed by increased nuclear PGC-1α, mitochondrial gene expression, and subsequent changes in mitochondrial protein content and enzyme activity during recovery (85, 86). These findings indicate that mitochondrial adaptation may start through modifications and activation of preexisting regulatory systems, rather than depending on an immediate rise in mitochondrial mass. With repeated exercise, this signaling response may be strengthened, as shown by stronger exercise-induced PGC-1α activation in trained than in untrained muscle (43). Accordingly, mitochondrial biogenesis is more appropriately understood as a dynamic sequence that begins with acute signaling events and is followed by longer-term structural and metabolic adaptation. Nevertheless, increased PGC-1α expression or nuclear abundance should be regarded as evidence of mitochondrial regulatory signaling rather than, by itself, proof of increased mitochondrial content or functional capacity.

The link between mitochondrial biogenesis and quality control is not entirely direct. Endurance exercise can raise indicators linked to mitochondrial biogenesis and fission, yet this does not necessarily match a prompt increase in mitophagy (46). In individuals who were trained and performed prolonged cycling, early post-exercise changes in mitophagy-related markers did not align with a broad activation of mitochondrial degradation, whereas nutritional status affected elements of signaling related to mitochondrial fission and biogenesis (46). These results suggest against viewing elevated expression of specific mitophagy-related proteins as proof that mitochondrial turnover has been completed. Rather, exercise-driven mitochondrial quality control may consist of processes that are separated in time, with their extent depending on exercise length, training condition, nutrient availability, and the recovery period that is assessed. This interpretation is supported by human skeletal-muscle studies showing that acute endurance exercise may increase fission-related signaling without producing a corresponding increase in conventional mitophagy markers, with responses differing according to fed versus fasted conditions (46). Conversely, more recent evidence suggests that resistance exercise can initiate mitochondrial remodeling and mitophagy-related structural changes, highlighting the importance of exercise modality and the distinction between mitophagy markers and mitophagic flux (87). Nutritional status is therefore not simply a background variable but a potential modifier of mitochondrial remodeling. Energy availability, carbohydrate availability, and the fed or fasted state may influence exercise-induced signaling, substrate use, mitochondrial dynamics, and recovery. This reinforces the broader framework of this review in which nutrition may act as a direct immunometabolic regulator, a modifier of exercise-induced signaling, and a determinant of substrate availability during adaptation rather than as a uniformly beneficial or detrimental factor.

This temporal dimension is especially important for immunometabolism. Remodeling of mitochondria in skeletal muscle could change the availability of metabolites, redox signals, and circulating mediators that can affect immune cells, yet the existing evidence does not demonstrate that muscle mitochondrial biogenesis directly leads to comparable mitochondrial adaptation in circulating leukocytes. A more defensible conclusion is that skeletal muscle is one element within a wider exercise-responsive metabolic network. Accordingly, the proposed muscle-to-immune connection should be considered an integrative model rather than a demonstrated linear pathway: direct evidence for muscle mitochondrial adaptation is substantially stronger than evidence showing that the same adaptation occurs in immune cells.

### 5.2 Mitochondrial metabolism and immune cell function

Direct investigations of immune cells are beginning to show evidence that adaptations linked to exercise may reach beyond skeletal muscle. After six weeks of either HIIT or moderate-intensity continuous training, several measures of mitochondrial bioenergetics were improved in human NK cells, including mitochondrial membrane potential, maximal and reserve oxygen consumption, and overall bioenergetic health (47). These effects coincided with increased levels of perforin and granzyme B, as well as a reduced mitochondrial oxidant load during follow-up exercise testing (47). Importantly, this randomized trial involved healthy young sedentary men, and therefore provides direct human evidence for exercise-associated NK-cell bioenergetic adaptation but limited evidence regarding older adults, women, or established immunosenescence. While these results are consistent with enhanced metabolic support for NK-cell effector function, the observational association between bioenergetic adaptation and cytotoxic capacity does not, on its own, demonstrate causation. Moreover, increased perforin and granzyme B should be interpreted as cellular functional markers rather than direct evidence of improved protection against infection or other clinical host-defense outcomes.

A similar pattern has been observed in lymphocytes subjected to hypoxic exercise stress. After six weeks of HIIT or moderate-intensity continuous training, lymphocytes showed increased oxygen consumption, higher citrate synthase and succinate dehydrogenase activity, an elevated mitochondrial membrane potential, and a reduced matrix oxidant burden during a hypoxic challenge (48). The rise in the mitofusin-to-DRP1 ratio also implies that mitochondrial dynamics shift toward a phenotype linked to improved mitochondrial integrity (48). However, the mitofusin-to-DRP1 ratio is an indirect indicator of mitochondrial dynamics and should not be interpreted as proof of improved mitochondrial quality or completed mitochondrial turnover. These results must be understood in the particular setting of hypoxic stress rather than generalized to all physiological conditions. Overall, they suggest that training may enhance lymphocytes’ ability to preserve mitochondrial function during metabolic stress, yet it is still not well established whether comparable effects arise during infection, chronic inflammation, or aging.

Acute exercise further clarifies the need to account for cellular composition and timing. Maximal exercise raised circulating PBMC counts, especially within particular CD8+ T-cell subpopulations, whereas tissue-level oxygen flux increased following exercise (49). By contrast, cellular OXPHOS and electron-transfer capacities did not show significant changes after being normalized to the PBMC count (49). Therefore, a seemingly increased whole-blood or tissue-level immune bioenergetic profile may be driven, at least in part, by the redistribution of metabolically distinct immune-cell populations rather than by an inherent enhancement of mitochondrial function within individual cells. This issue has also been observed after acute moderate-to-vigorous cycling, in which PBMC oxidative capacity increased at the tissue level without a corresponding increase in mitochondrial respiration on a cell-per-cell basis (88). Distinguishing between these possibilities is an important methodological concern for investigations of exercise immunometabolism. The cellular composition issue is particularly relevant when interpreting PBMC-based studies because PBMCs comprise metabolically heterogeneous populations of T cells, B cells, NK cells, and monocyte subsets. Acute exercise can preferentially mobilize particular phenotypes, including memory and senescent CD8+ T-cell populations, thereby altering the aggregate metabolic signal independently of intrinsic mitochondrial remodeling within each cell type (49).

Taken together, these findings indicate that exercise can affect immune-cell mitochondrial physiology, although the magnitude and direction of the response appear to vary depending on whether adaptation is evaluated acutely or after training, whether assessments are conducted at rest or under stress conditions, and whether outcomes are normalized at the cellular or whole-blood scale. This degree of variability may partly account for the apparently inconsistent results reported across exercise–immunometabolic studies. Exercise modality, training status, age, sex, metabolic health, nutritional state, and the timing of blood sampling should therefore be treated as important effect modifiers rather than secondary methodological details.

Sex-related biology also warrants greater consideration. Most direct training studies of immune-cell mitochondrial adaptation have been conducted in men, limiting generalizability to women and particularly to postmenopausal populations. Differences in sex hormones, menstrual or menopausal status, body composition, substrate utilization, and inflammatory responses may modify exercise-induced immune and mitochondrial responses (Klein & Flanagan, 2016; Gameiro et al., 2010). Future studies should therefore report sex-stratified analyses and, where relevant, hormonal or menopausal status rather than assuming that findings obtained predominantly in men apply uniformly across sexes.

### 5.3 Mitochondrial ROS, redox signaling, and inflammation

Mitochondrial ROS take up an intermediate role between metabolic adaptation and inflammatory signaling. Exercise does not merely suppress mitochondrial ROS; transient redox shifts also participate in cellular signaling and adaptation. Accordingly, the existing immune-cell evidence supports a more nuanced framework in which repeated exercise may strengthen the capacity to preserve mitochondrial redox homeostasis, rather than abolishing ROS production. This concept is consistent with the broader exercise-redox literature, in which transient and compartmentalized ROS signals can activate adaptive pathways involving AMPK, MAPK, PGC-1α, and antioxidant defenses, whereas excessive or sustained oxidant accumulation may instead contribute to cellular dysfunction (89, 90).

In natural killer cells, both HIIT and moderate-intensity continuous training were linked with a reduced mitochondrial matrix oxidant burden during an acute exercise challenge, alongside improved mitochondrial respiratory capacity (47). Likewise, trained lymphocytes exhibited lower mitochondrial oxidant burden during hypoxic exercise and higher activity related to oxidative phosphorylation (48). These results align with the idea that repeated exercise can increase mitochondrial robustness when immune cells confront an acute metabolic stressor. However, they do not establish that reduced mitochondrial ROS are universally responsible for improved immune function. Indeed, ROS should not be conceptualized solely as harmful by-products: their biological effects depend on dose, subcellular localization, timing, and redox-sensitive signaling pathways. Thus, a lower measured oxidant burden during a standardized stress test may indicate improved redox control in that experimental context without demonstrating that ROS reduction itself is the primary mechanism of immune adaptation.

Findings from macrophage models offer a mechanistically informative and complementary viewpoint. In mice, chronic moderate exercise was linked to enhanced mitochondrial quality, increased dependence on oxidative phosphorylation, and reduced mitochondrial reactive oxygen species production in bone-marrow-derived macrophages (50). These metabolic shifts coincided with diminished LPS-induced NF-κB activation and pro-inflammatory gene expression, along with changes in chromatin accessibility (50). These findings provide important preclinical evidence that exercise can produce persistent metabolic and epigenetic remodeling in macrophages, but they should not be directly extrapolated to human macrophage biology or interpreted as proof of improved host defense. The alignment of metabolic, redox, inflammatory, and epigenetic alterations supports the idea that exercise could modify macrophage inflammatory responsiveness via coordinated metabolic remodeling, rather than by suppressing a single inflammatory pathway. Notably, the phenotype described in this mouse model has been interpreted as “trained immunity”; however, the term should be used cautiously in this review because the durability, molecular mechanisms, and clinical consequences of such exercise-associated macrophage reprogramming remain incompletely defined in humans (50).

A related mechanism involves exercise-induced specialized proresolving mediators. Exercise training increased the production of proresolving lipid mediators in mice, whereas selected mediators enhanced mitochondrial respiration in murine and human macrophages via an AMPK-dependent mechanism (51). This supports a plausible pathway through which systemic exercise-derived signals could affect macrophage mitochondrial metabolism. However, because part of the supporting evidence comes from *ex vivo* mediator treatment and murine exercise models, the extent to which this pathway functions quantitatively in exercising humans remains uncertain. Accordingly, the findings are best considered mechanistic support for a possible exercise–lipid mediator–mitochondrial pathway rather than direct evidence of an established human anti-inflammatory mechanism.

Taken together, the evidence supports a model in which repeated exercise may shift immune-cell metabolism toward greater oxidative capacity and improved redox regulation in response to challenge. Such remodeling could contribute to changes in inflammatory responsiveness, but it should not be interpreted as a uniformly anti-inflammatory effect. The biological effects of mitochondrial reactive oxygen species are likely to depend on their magnitude, subcellular localization, timing, and cellular context. In particular, an exercise-induced reduction in inflammatory signaling is not necessarily equivalent to improved immune competence: appropriate inflammatory activation remains essential for pathogen recognition, tissue repair, and effective host defense.

### 5.4 Mitochondrial dysfunction in immune aging

Mitochondrial dysfunction is increasingly viewed as one element of the metabolic phenotype that accompanies immune aging. This phenotype is marked by disrupted energy metabolism, reduced stress adaptation, and alterations in immune-cell function and may intersect with changes in T-cell differentiation, NK-cell function, chronic innate activation, autophagy/mitophagy, and low-grade inflammation. Immunosenescence and inflammaging should nevertheless not be treated as synonymous processes: immunosenescence refers primarily to age-associated changes in immune-cell composition and function, whereas inflammaging describes a chronic, low-grade inflammatory state that may coexist with and contribute to immune dysfunction (14, 15). Accordingly, exercise-driven mitochondrial remodeling may offer a mechanistic basis for how physical activity could affect aspects of immunosenescence. However, compared with findings from younger or middle-aged cohorts, the direct evidence linking exercise-induced mitochondrial adaptation to the reversal or prevention of immune aging is still relatively limited. Mitochondrial dysfunction has been proposed as one component of age-associated immune decline, particularly in relation to altered T-cell metabolism, inflammatory signaling, and reduced cellular stress tolerance, but the causal contribution of mitochondrial changes to human immunosenescence remains incompletely resolved (91).

In the available human studies involving natural killer cells and lymphocytes, exercise training has been shown to enhance mitochondrial respiratory capacity, membrane potential, and redox regulation within immune cells (47, 48). Such changes may be important for aging, given that immune cells need to preserve metabolic flexibility during repeated activation and exposure to stress. Still, participants in these studies were typically generally healthy and relatively young or sedentary adults, rather than individuals with established immunosenescence. Accordingly, these findings should be interpreted as evidence for a potentially relevant biological pathway rather than direct evidence that exercise reverses age-associated mitochondrial dysfunction. This distinction is particularly important because the presence of a favorable mitochondrial phenotype in young or middle-aged adults does not establish that the same intervention will restore mitochondrial function in late-differentiated or senescent immune-cell subsets in older adults.

The macrophage results add an additional interpretive dimension. Exercise-related increases in oxidative phosphorylation, enhanced mitochondrial quality, and decreased mitochondrial ROS were accompanied by sustained changes in inflammatory responsiveness and chromatin accessibility in mouse macrophages (50). This indicates that metabolic remodeling can intersect with cellular programs that persist over longer timescales. It remains to be determined whether similar remodeling takes place in aged human macrophages, and whether it is sufficient to alter susceptibility to infection or chronic inflammatory disease. A key translational point is that mitochondrial adaptation may not confer uniform advantages across immune-cell subsets or disease contexts. Immune activation itself necessitates extensive metabolic reprogramming, and oxidative phosphorylation, glycolysis, mitochondrial ROS, and biosynthetic metabolism can play distinct roles depending on the cell’s activation state. Therefore, elevating mitochondrial respiratory capacity should not automatically be taken to reflect improved immune competence. The functional relevance of a mitochondrial adaptation depends on whether it enhances appropriate responses while preserving metabolic flexibility and limiting maladaptive inflammatory signaling.

From a clinical perspective, the most meaningful question is therefore not whether exercise increases a mitochondrial biomarker, but whether such cellular remodeling translates into preserved immune function in older or immunologically vulnerable populations. Relevant outcomes should include vaccine responsiveness, pathogen-specific immune responses, incidence or severity of clinically documented infections, infection duration, hospitalization, and other host-defense outcomes rather than relying exclusively on mitochondrial respiration, ROS, cytokines, or immune-cell phenotype. At present, direct evidence connecting exercise-induced immune-cell mitochondrial remodeling with these clinical outcomes remains limited.

The nutritional context should also be incorporated into this translational framework. Protein and amino-acid availability, carbohydrate availability, micronutrient status, energy sufficiency, and recovery nutrition may modify mitochondrial substrate availability and exercise-induced signaling. Conversely, low energy availability, recent illness, sleep disruption, metabolic disease, smoking, medication use, and circadian timing may alter both mitochondrial and immune responses. These factors should therefore be considered potential confounders or effect modifiers when interpreting observational and intervention studies.

Overall, existing evidence indicates that mitochondrial remodeling is a plausible element of exercise-driven immunometabolic adaptation; however, the evidentiary strength varies markedly across biological levels. Induction of mitochondrial signaling by exercise in skeletal muscle is relatively well supported (43–46), while direct evidence in immune cells is comparatively sparse and often relies on particular exercise or stress conditions (47–51). Taken together, the emerging picture does not reflect a single, universal exercise-related enhancement of mitochondrial function, but instead a context-dependent improvement in mitochondrial responsiveness, metabolic flexibility, and redox regulation (**Figure 1**, **Table 2**). The available evidence therefore supports a hierarchical interpretation: (i) direct human evidence is relatively strong for exercise-induced mitochondrial signaling and remodeling in skeletal muscle; (ii) direct human evidence exists for selected immune-cell bioenergetic adaptations, particularly in NK cells and lymphocytes, but is concentrated in healthy and relatively young populations; (iii) mechanistic evidence for macrophage metabolic and epigenetic reprogramming remains predominantly preclinical; and (iv) evidence linking these cellular adaptations to clinically meaningful host defense during aging remains limited. Determining whether these adaptations result in clinically meaningful preservation of immune competence during aging will necessitate longitudinal studies in older cohorts, analyses that distinguish specific immune-cell subsets, and interventions that combine exercise dose, nutritional status, mitochondrial phenotyping, and functional immune endpoints. Determining whether these adaptations result in clinically meaningful preservation of immune competence during aging will necessitate longitudinal studies in older cohorts, analyses that distinguish specific immune-cell subsets, and interventions that integrate exercise dose, nutritional status, mitochondrial phenotyping, and functional immune endpoints. Importantly, future studies should also determine whether exercise-associated changes in mitochondrial biomarkers translate into functional immune responses or clinically relevant host-defense outcomes.

**Figure 1:**
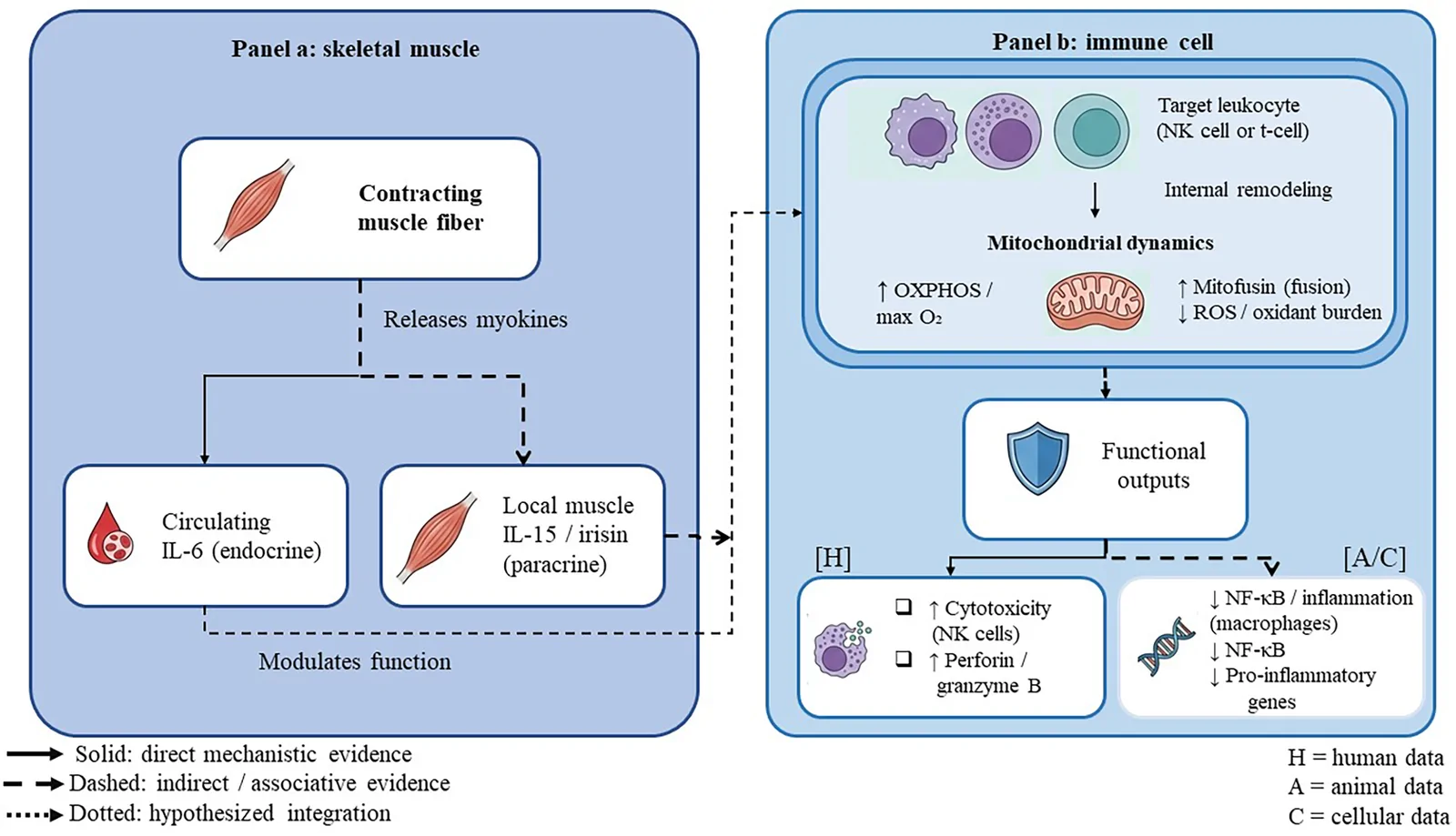
Skeletal muscle–immune crosstalk and exercise-associated mitochondrial adaptation. Exercise-induced skeletal muscle contractions can alter the production and release of muscle-derived signaling molecules, including IL-6 and IL-15, while some exercise-responsive mediators such as irisin may involve additional tissue sources. These signals may contribute to systemic immunometabolic communication and, depending on the exercise context and target tissue, may be associated with changes in immune-cell phenotype, metabolism, or function. The figure distinguishes different biological levels of evidence and does not imply that circulating myokine concentrations necessarily demonstrate secretion from skeletal muscle, receptor engagement, or downstream immune-cell effects. Mitochondrial responses are represented at distinct levels, including mitochondrial biogenesis-related signaling, respiratory function, redox regulation, mitochondrial dynamics, and mitophagy, which should not be considered interchangeable measures of mitochondrial adaptation. Evidence obtained directly in humans, including exercise-associated changes in selected immune-cell functional or bioenergetic outcomes, is distinguished from mechanistic evidence derived from animal or *in vitro* models. Solid arrows represent relationships supported by direct human or experimentally established mechanistic evidence, whereas dashed arrows indicate indirect, associative, extrapolated, or preclinical/*in vitro* relationships for which direct causal evidence in humans remains limited. Accordingly, the figure presents a graded model of exercise–muscle–immune communication rather than a fully validated linear pathway from myokine release to immune-cell mitochondrial remodeling.

## 6 Nutritional modulation of exercise-induced immunometabolic adaptation

Nutritional availability is a key contextual factor shaping the immune and metabolic responses to exercise, yet the existing evidence does not indicate a consistent immunoprotective effect from any single dietary approach. Instead, nutritional regulation seems to depend on and interact with exercise intensity, duration, training status, baseline nutritional condition, as well as the timing and composition of nutrient intake. Accordingly, nutrition is considered here in three partially overlapping roles: as a direct determinant of nutrient and energy availability for immune-cell metabolism, as a modifier of exercise-induced signaling and mitochondrial adaptation, and as a determinant of recovery from the energetic and physiological demands imposed by training. These effects are likely to be context-dependent rather than uniformly beneficial. Across human intervention studies, the most reliable finding is that dietary manipulation can alter particular physiological, mucosal, oxidative, or inflammatory markers, whereas impacts on wider immune function are less consistent (**Table 3**). This distinction matters because changes in individual biomarkers should not automatically be taken as proof of enhanced immune competence or diminished clinical risk of infection. In particular, changes in cortisol, cytokines, salivary IgA, oxidative-stress markers, or circulating leukocyte counts should be distinguished from cellular functional outcomes, vaccine responses, pathogen-specific immunity, clinically documented infection, infection duration or severity, and hospitalization. Direct evidence linking nutritional manipulation during exercise with these clinically meaningful host-defense outcomes remains limited.

### 6.1 Macronutrient availability and immune metabolism

Carbohydrate availability seems especially important during prolonged or intensified endurance exercise, a time when energetic demands and neuroendocrine stress are elevated. Raising carbohydrate intake during intervals of increased training load has been linked to reduced magnitude of certain stress-related responses and to preservation of salivary immune markers. Among trained triathletes, a brief phase of high-carbohydrate consumption during a period of increased exercise workload corresponded to higher post-exercise salivary IgA and a cortisol response that was less pronounced than that observed with a self-selected diet (52). Nevertheless, these results should be considered alongside the specific exercise model and the relatively brief intervention period, and they do not demonstrate that greater carbohydrate intake prevents infection associated with exercise. More broadly, carbohydrate availability appears to influence the magnitude of physiological stress responses particularly during prolonged, glycogen-demanding exercise, but this should not be interpreted as evidence that carbohydrate supplementation uniformly improves immune competence.

Conversely, deliberately limiting carbohydrate availability does not seem to trigger a consistent worsening of measured immune responses when applied over a brief period under controlled training and nutritional conditions. Among elite triathletes, implementing a short-term “sleep-low” approach resulted in acute IL-6 responses that were comparable to those seen with higher carbohydrate availability, whereas salivary IgA was largely unaffected; however, hepcidin responses were higher after one low-carbohydrate condition (53). Overall, these findings indicate that carbohydrate periodization may selectively affect metabolic and iron-regulatory pathways without necessarily producing a broad decline in immune markers. The variability across studies may be partly attributable to differences in exercise duration, carbohydrate dose, the timing of intake relative to exercise, training status, and the specific immune compartment assessed. Importantly, carbohydrate restriction should also be distinguished from inadequate total energy availability. Low energy availability represents a broader physiological state in which energy intake is insufficient relative to exercise expenditure and other physiological requirements and can affect multiple systems, including endocrine, metabolic, reproductive, bone, and immune physiology (92).

Importantly, high carbohydrate intake does not seem to consistently change immune responses across different exercise modalities. After resistance exercise, consuming carbohydrate did not significantly affect salivary IgA, IL-2, or the exercise-related decrease in IL-5 (55). Therefore, the potential benefit of carbohydrate availability may be more apparent during prolonged endurance exercise or under marked energetic stress than following shorter, resistance-based protocols. Taken together, these results indicate a context-dependent rather than universally immune-enhancing role for carbohydrate availability. This distinction is particularly relevant when comparing endurance, interval, and resistance exercise, because the magnitude of glycogen depletion, catecholamine and cortisol responses, muscle mass recruited, and recovery demands differs substantially among these modalities.

### 6.2 Protein, amino acids, and muscle–immune signaling

Protein and amino-acid availability may provide another point of contact between skeletal muscle metabolism and immune regulation. Glutamine has long been suggested as a potential mediator, since strenuous exercise can transiently change circulating glutamine availability and lymphocyte responses. Nevertheless, controlled experimental data offer only modest support for a predominant causal function. Supplementing with glutamine during and after prolonged cycling prevented the expected decrease in circulating plasma glutamine, but it did not meaningfully affect lymphocyte trafficking after exercise, natural killer-cell activity, lymphokine-activated killer-cell activity, or T-cell proliferation (54). This separation between the recovery of substrate availability and the behavior of immune cells indicates that short-lived variations in circulating glutamine are unlikely, on their own, to account for the acute immune alterations seen following prolonged exercise. Consistent with this interpretation, controlled trials summarized in systematic evidence have generally not demonstrated a consistent improvement in immune outcomes following glutamine supplementation in exercising populations (93).

Longer-term protein supplementation might yield more targeted effects. In a resistance-training intervention, providing hydrolyzed beef protein, whey protein, or carbohydrate resulted in relatively modest between-group differences in salivary human neutrophil peptides; however, HNP1–3 concentration and secretion declined over time in the beef-protein group (56). Taken together, these results imply that the protein source may affect specific mucosal immune markers, yet they do not demonstrate a broad improvement in immune function. Variations in protein dose, amino-acid composition, energy availability, training adaptation, and the immune outcome that was assessed are likely to account for the differences observed across responses.

An important distinction is therefore needed between meeting protein requirements and using protein or amino-acid supplements specifically to modify immunity. Adequate protein intake supports tissue remodeling, muscle protein turnover, and recovery, whereas evidence that additional protein supplementation independently enhances systemic immune competence in well-nourished exercising adults remains inconsistent. A systematic review of human trials found weak and heterogeneous effects of protein or amino-acid supplementation on post-exercise inflammatory and oxidative-stress markers, with most included studies reporting no significant effect on these outcomes (94).

From a mechanistic standpoint, these findings suggest that the muscle–immune interface is unlikely to be controlled by the availability of any single amino acid or nutrient. Rather, protein availability may shape immune metabolism indirectly by supporting muscle protein turnover, promoting tissue repair, affecting substrate availability, and modulating exercise-induced signaling. Translationally, this favors an emphasis on adequate overall protein and energy availability rather than supplementation with individual amino acids in the absence of a demonstrated nutritional deficiency. This interpretation is also consistent with the broader concept that immune cells require substantial metabolic substrates during activation, while the relative contribution of glucose, glutamine, fatty acids, and other substrates varies according to immune-cell type and activation state (95).

### 6.3 Dietary lipids and inflammatory signaling

Dietary fatty acids may influence inflammation associated with exercise by altering membrane lipid composition and by promoting the production of lipid-derived signaling mediators. Recent findings using docosahexaenoic acid (DHA) lend partial support to this idea. Twelve weeks of DHA supplementation raised the omega-3 index and coincided with reduced delayed-onset muscle soreness after eccentric exercise, and differences in the post-exercise responses of IL-6, TNF-α, and IL-10 were also reported (60). Nevertheless, cytokine interaction effects should not be interpreted as evidence of a consistently anti-inflammatory response, and the study does not demonstrate that DHA directly reprograms immune-cell metabolism.

This distinction is especially important because exercise-induced inflammation is not inherently pathological. Acute inflammatory signaling helps drive tissue remodeling and adaptation, and suppressing these responses to an excessive degree may not necessarily be advantageous. Therefore, the translational relevance of dietary lipid supplementation may lie less in simply reducing inflammatory biomarkers and more in modulating the magnitude, timing, and resolution of exercise-induced inflammatory responses without necessarily abolishing adaptive signaling. Evidence remains insufficient to determine whether such effects translate into meaningful improvements in long-term immune health or training adaptation. Moreover, changes in circulating cytokines should not be interpreted as direct evidence of altered immune-cell function unless accompanied by cellular or functional measurements.

### 6.4 Micronutrients and redox pathways

Antioxidant micronutrients illustrate, in a clear way, the complexity involved in how nutrition modulates exercise-induced immunometabolic responses. Physical activity increases the production of reactive oxygen species, yet these molecules also act as signaling intermediates that participate in metabolic adaptation. Therefore, lowering oxidative biomarkers does not necessarily correspond to better physiological adaptation. Supplementation trials in humans using vitamin E, or a combination of vitamin C and E, have yielded mixed results. After eight weeks of vitamin E supplementation, malondialdehyde, protein carbonyls, creatine kinase, and exercise performance following exhaustive exercise did not change significantly (57). Likewise, combined antioxidant supplementation has been linked to decreases in selected oxidative-stress or muscle-damage markers, although responses differed across individual biomarkers and between supplementation groups (58). In a larger study of exercise training, vitamin E supplementation did not lead to clear additional effects on oxidative-stress or inflammatory markers relative to placebo (59). Taken together, these findings do not support a consistent effect of high-dose vitamin E or vitamin C/E supplementation on exercise-induced oxidative or inflammatory responses.

Evidence regarding antioxidant supplementation and training-related adaptation is similarly inconsistent. In some human studies, combined vitamin C and E supplementation has been reported to reduce exercise-associated mitochondrial or metabolic adaptations (96), while other well-controlled investigations have observed no substantive disruption to endurance-training adaptations (97). Taken together, these conflicting results highlight the need to consider supplement dosage, baseline nutritional status, the exercise protocol, training duration, and the particular biological endpoint being measured, rather than concluding that antioxidant supplementation has a uniform effect.

The observed heterogeneity may arise from differences in baseline antioxidant status, supplement dose, intervention duration, the exercise stimulus, sex, training status, and both the timing and sensitivity of the oxidative-stress measurements. More fundamentally, the findings emphasize that exercise-induced redox signaling should not be regarded only as a detrimental process that ought to be suppressed. Nutritional interventions that substantially shift redox balance could, in theory, affect both harmful and adaptive signaling pathways, even though the clinical importance of this possibility remains incompletely established. Sex and age should also be considered when interpreting micronutrient studies because antioxidant status, hormonal environment, metabolic health, and habitual dietary intake may modify both redox responses and training adaptation.

Polyphenol-rich foods offer a related yet distinct nutritional approach. A recent randomized trial reported that a brief polyphenol-rich food intervention substantially increased circulating polyphenol metabolites, while showing only limited and inconsistent effects on early post-exercise inflammatory and oxidative-stress markers (61). Notably, some oxidative biomarkers did not change in the direction that a straightforward antioxidant model would predict. These findings further suggest that dietary bioactives should not be characterized solely according to their capacity to lower oxidative-stress biomarkers; their biological effects are likely to depend on metabolism, dose, food matrix, timing, and the specific exercise-induced redox environment. Importantly, this type of intervention provides evidence for altered exposure to dietary polyphenol metabolites rather than direct evidence of improved immune function. A recent randomized controlled trial similarly found increased circulating polyphenol metabolites after a short polyphenol-rich food intervention but limited and inconsistent effects on post-exercise inflammatory and oxidative-stress markers (61).

### 6.5 Nutritional timing and exercise-induced immune adaptation

How nutrients are made available in relation to exercise may matter as much as their composition. Findings from carbohydrate periodization suggest that training in settings of low carbohydrate availability can alter certain metabolic and iron-regulatory responses, yet without yielding consistent changes in standard immune markers (53). Likewise, consuming carbohydrate immediately before, during, and after resistance exercise did not meaningfully change the salivary immune response that was measured (55). Together, these results indicate that the impact of nutritional timing depends heavily on the physiological context in which the exercise stimulus takes place.

A broader interpretation is that nutritional timing may affect the size of exercise-induced immunometabolic stress and the pattern of recovery, rather than acting as a straightforward switch for immune function. Sufficient carbohydrate availability may be especially important when the volume and duration of exercise create considerable energetic strain, whereas intentional nutrient restriction may be better tolerated when it is implemented selectively within an otherwise adequate nutritional plan. However, deliberate carbohydrate restriction should not be equated with chronic low energy availability, and neither strategy should be considered inherently advantageous without accounting for total energy intake, training load, body composition, health status, and recovery demands. Protein timing may similarly interact with muscle recovery and substrate availability, but current evidence does not demonstrate that specific timing strategies consistently enhance systemic immune function.

Nutritional timing may also affect circadian biology. The timing of meals, exercise, sleep, and circadian phase can shape metabolic and inflammatory physiology, yet there is still insufficient direct evidence that altering these factors enhances immune competence related to exercise. Consequently, timing should currently be viewed as a possible modifier that warrants further study rather than a clinically established approach for improving immunity.

Overall, the evidence available indicates a framework in which nutrition functions as a modifier of exercise-driven immunometabolic reorganization, rather than as an independent determinant of immune adaptation. The availability of macronutrients, protein intake, dietary fatty acids, and consumption of foods rich in antioxidants can each affect particular parts of the response, yet the effects are heterogeneous and frequently depend on the specific biomarker. The main contributors to this heterogeneity include the type and dose of exercise, training status, sex, baseline diet and nutritional status, length of the intervention, the dose and composition of the nutrient, the timing of intake in relation to exercise, and variations across circulating, mucosal, and cellular immune outcome measures. Additional sources of heterogeneity include age, metabolic health, adiposity, habitual physical activity, sleep, psychological stress, medication use, recent infection or vaccination, energy availability, and potentially the gut microbiome. These factors may act either as confounders or as effect modifiers and should therefore be characterized in future nutritional exercise studies. These considerations should guide how nutritional results from controlled exercise studies are translated into practical recommendations.

From a translational standpoint, the existing evidence thus supports ensuring sufficient energy and macronutrient intake matched to the demands of training, rather than routinely using high-dose supplements aimed at reducing exercise-induced inflammation or oxidative stress. This should be interpreted as an evidence-informed general principle rather than as a universal prescription, because individual nutritional requirements vary with age, sex, training load, body composition, health status, and baseline dietary adequacy. Notably, the lack of consistent immune advantages from glutamine or antioxidant supplementation argues against treating a normalization of a single biomarker as proof of enhanced immune resilience (54, 57–59). Future research should link dietary exposure with cellular immunometabolism, mitochondrial function, inflammatory resolution, and clinically meaningful outcomes, while also considering baseline nutritional status and the amount of exercise performed. Priority outcomes should include functional immune-cell responses, vaccine responsiveness, pathogen-specific immunity, and clinically documented infection incidence, duration, severity, or hospitalization, particularly in older adults and other immunologically vulnerable populations. This framework may help clarify whether nutritional interventions can meaningfully shape long-term immune adaptation and immune aging, instead of simply changing short-lived biomarker levels following separate exercise sessions.

## 7 Exercise, immunosenescence, and inflammaging

### 7.1 Cellular and molecular hallmarks of immune aging

Immune aging is defined by a gradual reorganization of immune-cell populations rather than by a uniform reduction in immune function. The adaptive compartment is especially affected, with reductions in naïve T-cell pools, accumulation of differentiated and senescence-associated T-cell phenotypes, alterations in the CD4+/CD8+ ratio, and changes in proliferative and antigen-specific responsiveness. These changes may coexist with altered cytokine production and reduced responsiveness to newly encountered antigens, but their magnitude and functional consequences vary substantially among individuals. By contrast, some components of innate immunity may remain relatively preserved, whereas others may exhibit altered activation or chronic inflammatory signaling, contributing to a heterogeneous immune phenotype in older adults (14). Accordingly, immunosenescence should be understood as a multidimensional remodeling process involving cellular composition, differentiation, immune regulation, and functional responsiveness rather than as a simple loss of immune activity.

Chronological age alone cannot explain this heterogeneity. Exposure to cytomegalovirus (CMV), cumulative antigenic history, sex and hormonal status, adiposity, physical fitness, lifestyle, and comorbidities can influence the distribution and functional characteristics of immune-cell subsets. In a large population-based analysis, naïve CD4+ and CD8+ T-cell counts decreased with increasing age, whereas CMV serostatus was associated with distinct patterns of T-cell composition (98). Likewise, studies in very old adults indicate substantial interindividual variation in highly differentiated T-cell subsets and CD4+/CD8+ ratios (99). CMV appears particularly relevant to the accumulation of highly differentiated effector-memory and TEMRA phenotypes, although its relationship with naïve T-cell depletion is less consistent across studies (100). Sex-related biological differences may also contribute to immune-aging trajectories through differences in sex hormones, body composition, immune-cell distributions, and inflammatory responses; these factors become particularly relevant during and after menopause (19, 20).

Importantly, immunosenescence and inflammaging are related but non-synonymous processes. Immunosenescence primarily refers to age-associated remodeling and functional alteration of immune-cell compartments, whereas inflammaging describes a persistent, low-grade inflammatory state involving immune and non-immune tissues (14, 15). An individual may therefore exhibit substantial remodeling of T-cell or NK-cell compartments without parallel changes in conventional circulating inflammatory biomarkers. This distinction is particularly important when interpreting exercise studies because a change in one cellular or molecular marker does not establish reversal of immune aging or improvement in host defense.

### 7.2 Exercise-induced modulation of immunosenescence

Exercise may influence several characteristics associated with immune aging, but the available evidence does not support the conclusion that regular exercise uniformly reverses immunosenescence. A systematic review of exercise effects on cellular immunosenescence markers concluded that findings remain heterogeneous and are strongly influenced by exercise type and intensity, with long-term evidence in older adults still relatively limited (18).

A recurring observation is that exercise can modify the distribution of circulating T-cell subsets, including naïve, memory, and more differentiated populations. In older women, a six-week strength-endurance intervention decreased circulating senescence-prone T-cell populations, with the effect observed predominantly among CMV-seropositive participants (101). This finding illustrates that exercise responsiveness may depend on pre-existing immune history rather than exercise exposure alone. Other intervention studies have reported changes in naïve, central-memory, effector-memory, or TEMRA populations following regular exercise, but the direction and magnitude of these responses vary according to modality, intensity, intervention duration, baseline activity, age, sex, CMV status, and the immunophenotypic markers selected. Exercise-induced changes in immune-cell phenotypes may also differ according to training modality and intensity. However, available intervention studies in older adults remain heterogeneous, and current evidence does not support a uniform immunological response across moderate continuous, resistance, combined, or high-intensity interval exercise (18).

Acute exercise should also be distinguished clearly from chronic training adaptation. The transient mobilization of differentiated, memory, or other lymphocyte subsets during and immediately after exercise primarily reflects redistribution between blood and peripheral tissues and should not automatically be interpreted as depletion or expansion of those populations. This temporal distinction is particularly important because acute exercise can produce large but short-lived changes in circulating cell counts, whereas chronic training is more appropriately evaluated through changes in resting immune-cell distributions or repeated functional measurements (9, 18).

By contrast, repeated exercise may modify resting immune-cell phenotypes, although the durability and functional consequences of these changes remain uncertain. Findings in older adults suggest that habitual physical activity and higher physical fitness may be associated with more favorable immune-cell profiles, including aspects of NK-cell and T-cell regulation (102). However, such associations cannot by themselves establish that exercise restores immune function to a younger state, particularly because observational studies may be affected by reverse causation and healthy-participant bias. Recent three-year longitudinal evidence in clinically healthy older adults further showed that declines in physical fitness were accompanied by shifts toward more differentiated and senescence-associated T-cell phenotypes, while conventional circulating pro-inflammatory cytokines remained relatively stable (103). This observation reinforces the need to distinguish immune-cell remodeling from systemic inflammatory status.

The functional significance of these cellular changes also requires careful qualification. A higher proportion of naïve or central-memory cells, a lower frequency of senescence-associated phenotypes, or altered NK-cell abundance is a molecular or cellular endpoint; none should be interpreted as equivalent to improved vaccine effectiveness, enhanced pathogen-specific immunity, reduced infection incidence, or lower infection severity unless these outcomes are directly assessed.

### 7.3 Inflammaging and chronic low-grade inflammation

Inflammaging describes a persistent, generally low-grade inflammatory state that becomes more prevalent with advancing age and involves dysregulated control of inflammatory cytokines, acute-phase proteins, immune-cell activity, and tissue-derived inflammatory signals. Rather than representing a single pathway, inflammaging appears to emerge from interactions among immune-cell aging, persistent antigenic stimulation, cellular damage, adipose-tissue inflammation, metabolic dysfunction, altered tissue homeostasis, and other age-related processes (14, 15).

Exercise is frequently associated with lower circulating concentrations of selected inflammatory biomarkers in older adults, but the magnitude and consistency of these effects vary. Meta-analyses of exercise interventions in older adults have reported reductions in markers such as IL-6, TNF-α, and CRP, although heterogeneity across exercise modalities, participant health status, and study protocols remains substantial (104). Therefore, reductions in CRP, IL-6, or TNF-α should be interpreted primarily as changes in molecular inflammatory biomarkers rather than as direct evidence of restored immune competence.

This distinction is particularly important because inflammatory biomarkers are influenced by adiposity, metabolic disease, medication use, physical fitness, habitual activity, sleep, psychosocial stress, recent infection or vaccination, and the timing of blood sampling relative to the last exercise bout. Acute exercise itself can transiently increase IL-6 and other signaling molecules as part of a physiological response to exercise, which is mechanistically distinct from persistent low-grade inflammation. Thus, the timing of biomarker assessment and the distinction between acute signaling and chronic resting-state adaptation are essential for interpretation. Indeed, exercise training does not consistently modify every inflammatory biomarker, even when physical fitness or physical function improves. Recent evidence in older adults with frailty or sarcopenia, for example, indicates that effects may differ among TNF-α, IL-6, and CRP rather than showing a uniform anti-inflammatory response (105). Such findings argue against treating any single inflammatory biomarker as a surrogate for the overall state of inflammaging. The relationship between immune remodeling and inflammaging is further illustrated by longitudinal data showing that age-related changes in T-cell phenotypes can occur despite relatively stable concentrations of conventional pro-inflammatory cytokines (103). Thus, exercise may influence inflammatory regulation and immune-cell composition through partially overlapping but not identical biological pathways.

Importantly, the clinical relevance of these molecular changes remains incompletely established. Evidence linking exercise-induced changes in inflammatory biomarkers specifically to clinically meaningful host-defense outcomes in older adults including infection incidence, infection severity or duration, hospitalization, and protection following vaccination, is considerably less extensive than evidence based on circulating biomarkers. Some evidence suggests that habitual physical activity or exercise can influence antibody responses to influenza vaccination, but effects are heterogeneous and depend on age, exercise characteristics, baseline activity, and study design (106). Accordingly, biomarker improvements should not be presented as equivalent to demonstrably improved resistance to infection.

### 7.4 Reversibility and limitations of exercise-induced immune remodeling

A key unresolved question is whether exercise-associated immune remodeling represents durable reversal of immunosenescence or instead a partial, phenotype-specific, and context-dependent modification of age-associated immune characteristics. Current evidence is more consistent with the latter interpretation. In a 12-week structured exercise intervention in older adults, expression of several senescence-associated markers, including p16, p21, cGAS, and TNF-α, was reduced in circulating CD3+ T cells, accompanied by lower circulating concentrations of several senescence-related proteins (107). These findings provide evidence that some biomarkers associated with cellular senescence are modifiable in humans, but they do not establish reversal of immunosenescence, restoration of biological age, or improved protection against infectious disease. Moreover, interpretation of these findings should remain cautious because the study assessed molecular markers rather than comprehensive immune-cell function or clinical host-defense outcomes. This distinction is especially important because canonical senescence markers such as p16 and p21 are molecular indicators rather than direct measures of immune competence. Similarly, circulating senescence-associated proteins may reflect systemic biological aging but cannot, in isolation, establish functional immune restoration. Recent systematic evidence also indicates that the literature remains insufficiently standardized for determining whether exercise consistently modifies canonical cellular-senescence pathways in older adults, underscoring the need for studies combining upstream senescence markers with cellular function and clinical outcomes.

The persistence of exercise-associated immune adaptations also remains uncertain, particularly following cessation of training or substantial reductions in training volume. Moreover, variability in participant characteristics limits generalizability. CMV serostatus, sex and menopausal status, age, baseline fitness, training history, adiposity, metabolic health, medication use, comorbidities, sleep, nutritional status, recent infection or vaccination, and habitual physical activity may all modify immune responses to exercise. These factors should therefore be considered potential effect modifiers or confounders rather than secondary details. Exercise interventions also vary substantially in modality, intensity, frequency, duration, recovery interval, and training status, making direct comparison difficult. Aerobic/endurance, resistance, combined, and interval exercise should not be assumed to produce interchangeable immunological effects, and acute responses should not be extrapolated directly to chronic adaptations. Similarly, findings obtained in healthy older adults may not generalize to individuals with obesity, diabetes, frailty, chronic inflammatory disease, or immunological vulnerability. Sex-related heterogeneity merits particular focus. Women and men can differ in distributions of immune cells, inflammatory responses, substrate utilization, body composition, and endocrine regulation, and menopause adds another shift in exposure to sex hormones that may affect immune and inflammatory phenotypes (19, 20). Accordingly, future trials should report outcomes stratified by sex when sufficiently powered, and they should take menopausal and hormonal status into account when interpreting exercise-related immune remodeling. The translational evidence should also be organized across multiple tiers: molecular biomarkers, cellular phenotypes, functional immune responses, vaccine responses, pathogen-specific immunity, and clinical infection outcomes. Findings at one tier should not be assumed to constitute evidence at the next. For example, a reduction in a senescence-associated marker or an increase in a naïve T-cell proportion does not necessarily demonstrate improved immune-cell function, enhanced vaccine responsiveness, or reduced infection risk.

Taken together, exercise is best viewed as a potentially modifiable determinant of selected features of immune aging rather than as a proven intervention that globally reverses immunosenescence. Future longitudinal studies should integrate multidimensional immune phenotyping with functional assays and clinically meaningful endpoints, including vaccine responsiveness, pathogen-specific immune responses, infection incidence and severity, and hospitalization where appropriate. Such studies should also determine whether observed cellular and molecular changes persist after training cessation and whether they are mediated by exercise modality, dose, fitness adaptation, nutritional status, metabolic health, or other host characteristics. This framework may help determine whether exercise-associated immune remodeling represents transient biological adaptation, sustained immune resilience, or clinically meaningful preservation of host defense.

## 8 Exercise-induced immunometabolic reprogramming and host defense

Exercise triggers a coordinated physiological response involving metabolic, endocrine, vascular, and immune pathways. Rather than causing a uniform increase or decrease in immune function, available evidence indicates that exercise can temporarily redistribute immune cells, alter inflammatory signaling, and affect components of systemic and mucosal host defense. However, these responses occur at different biological levels and should not be interpreted interchangeably: changes in circulating immune-cell numbers or molecular biomarkers do not necessarily indicate enhanced immune-cell function, improved vaccine responsiveness, or protection against infection. The direction and magnitude of these effects appear to depend on exercise dose, duration, training status, age, sex, baseline immune function, nutritional and metabolic state, recovery, and the nature and timing of the immune challenge. Accordingly, exercise-related immunometabolic adaptation is more accurately viewed as a context-dependent process than as a consistently immunostimulatory phenomenon (**Table 4****).** This distinction is particularly important in older adults, in whom immune aging, CMV serostatus, adiposity, metabolic disease, medication use, sleep, psychological stress, and habitual activity may modify exercise–immune associations (64).

### 8.1 Innate immune defense and barrier function

Acute exercise can cause substantial changes in the trafficking of innate immune cells. For example, in older adults, acute resistance and cardiorespiratory exercise produced partly distinct patterns of T-cell mobilization in physically active versus inactive individuals, illustrating that the same exercise stimulus does not necessarily produce the same cellular response across populations (64). These acute changes primarily represent redistribution among blood, lymphoid, and peripheral tissue compartments and should therefore not be interpreted as evidence of increased immune-cell production or improved immune competence.

Mucosal immune protection may be particularly responsive to exercise load. Secretory immunoglobulin A (sIgA) is an important component of upper-airway mucosal defense, and experimental studies have reported transient reductions in salivary sIgA following sufficiently intense or prolonged exercise (69). In healthy young men, exercise at ≥75% VO_2_max for 30 min or exercise for 90 min at 55% VO_2_max reduced sIgA secretion immediately after the exercise bout (69). Likewise, prolonged training combined with hypoxic exposure was associated with a persistent reduction in salivary IgA among elite endurance athletes (70). A similar cumulative reduction in sIgA was reported during an 18-day “live-high/train-low” intervention, with incomplete recovery after two weeks, suggesting that environmental stressors such as hypoxia may modify the mucosal response to exercise (108). Overall, these findings indicate that exercise-related alterations in mucosal immunity may depend strongly on physiological stress, training volume, environmental exposure, and recovery. Importantly, reduced post-exercise sIgA is a mucosal biomarker rather than a direct measure of clinical infection susceptibility. Although lower sIgA has been associated with respiratory illness in some athlete populations, the causal contribution of exercise-induced changes in sIgA to infection risk remains uncertain (109, 110).

Exercise-related changes in inflammatory signaling may likewise reflect context-dependent regulation rather than generalized immune suppression. During exercise-associated increases in core temperature, alterations in leukocyte trafficking have been accompanied by changes in inflammatory cytokine responses, including attenuated TNF-α and IFN-γ responses at higher core temperatures and increased LPS-stimulated IL-10 production during exercise (111). These findings are consistent with the possibility that exercise-induced inflammatory responses may be accompanied by counter-regulatory mechanisms that limit excessive inflammatory activation under physiological stress (111–113). Nevertheless, altered cytokine production should be considered a molecular or cellular response rather than a direct indicator of improved host defense. In particular, acute exercise-induced IL-6 and other cytokine responses should not be equated with chronic anti-inflammatory effects or enhanced resistance to infection. Thus, the immunological effects of exercise may be better interpreted by considering both the magnitude of immune activation and the concurrent regulation of potentially harmful inflammation (111–113).

### 8.2 Adaptive immunity and immune surveillance

Exercise can affect adaptive immune compartments through both short-term redistribution and longer-term training-related adaptations. Acute cardiorespiratory and resistance exercise mobilize differentiated CD8+ T-cell populations and yield different responses in physically active versus inactive older adults (64). These observations provide evidence for exercise-induced immune-cell redistribution, but they do not establish that the mobilized cells have greater antigen-specific functionality or that tissue-level immune surveillance is improved. Higher numbers of immune cells in peripheral blood therefore do not necessarily reflect enhanced immune function at the tissue level or increased resistance to infection.

Longer-term exercise may produce more enduring effects on selected components of adaptive immunity, especially in older adults. Moderate exercise training has been linked to larger increases in antibody responses to influenza vaccination and higher Granzyme B activity (62). A separate long-term aerobic exercise intervention in older adults was also associated with strengthened antibody and IFN-γ responses after influenza vaccination (73). For example, a 10-month cardiovascular exercise intervention in sedentary older adults increased longer-term influenza vaccine seroprotection for some vaccine strains, although peak antibody titers were not consistently increased (62). Nevertheless, these findings have not been consistently replicated.

In the MEPARI trial, neither exercise nor mindfulness-based stress reduction significantly enhanced influenza vaccine responses compared with controls (71). Similarly, a randomized trial of a 16-week lifestyle physical-activity intervention in sedentary women did not demonstrate a significant improvement in antibody responses to pneumococcal vaccination, despite increased physical activity (114). As a result, regular exercise might help maintain particular components of adaptive immune responsiveness in some populations, but the present evidence does not support a universal improvement in vaccine-induced immunity.

Another important consideration is immunological aging. In older adults, regular combined exercise has been associated with differences in inflammatory markers and CD8+ T-cell profiles, as well as changes in immune markers related to CMV, in relation to influenza vaccination responses (67). These findings are consistent with the possibility that habitual exercise can modify selected characteristics associated with immunosenescence. However, exercise-associated changes in senescence-related cellular phenotypes should be distinguished from restoration of naïve-cell pools, thymic function, antigen-specific immune competence, or clinically meaningful host defense. Moreover, because exercise behavior is closely tied to other health and lifestyle factors, such associations should not be taken as conclusive evidence of causation. Reverse causation and healthy-participant bias are particularly relevant in observational studies, because individuals with better baseline health may be more likely to maintain regular physical activity.

### 8.3 Exercise and antiviral/antimicrobial responses

The potential relevance of exercise-induced immune modulation to host defense has been investigated most directly through vaccination studies, but vaccine responsiveness represents an intermediate functional outcome and should not be considered equivalent to protection from naturally acquired infection. Acute exercise has occasionally been shown to enhance antibody responses when performed around the time of vaccination. For instance, a short exercise intervention before pneumococcal vaccination produced greater antibody responses, with the effect particularly evident after a lower vaccine dose (63). In a randomized trial of 133 young adults, 15 min of upper-body exercise before pneumococcal vaccination increased antibody responses, with a larger exercise-associated effect observed after the half-dose vaccine than after the full-dose formulation (63). Likewise, a 90-min bout of light-to-moderate aerobic exercise performed after vaccination was associated with higher serum antibody responses to influenza and COVID-19 vaccines (65). This study therefore supports the possibility that exercise can function as a behavioral adjuvant under selected experimental conditions, but the finding was obtained under a specific exercise dose, timing, and vaccination protocol and should not be generalized to routine clinical vaccination.

However, this effect is not observed consistently. Acute eccentric resistance exercise performed before influenza vaccination did not enhance antibody levels or cell-mediated responses when the workout occurred immediately, 6 h, or 48 h before vaccination (72). Similarly, acute eccentric resistance exercise immediately before influenza vaccination did not improve antibody titers or influenza-specific T-cell responses in older adults (68). More recent evidence also indicates that a single 45-min moderate-intensity resistance exercise bout before influenza vaccination in older adults reduced vaccine-related reactions but did not alter antibody responses or influenza-like illness during six months of follow-up. The differences between these studies indicate that exercise-related immunomodulation cannot be explained simply by whether physical activity is present or absent. Exercise modality, intensity, duration, timing relative to vaccination, vaccine formulation, participant characteristics, prior immune exposure, and baseline immune responsiveness may all affect the outcome. A systematic review with individual-participant-data meta-analysis found that acute exercise and habitual physical activity were associated with some higher influenza vaccine antibody responses, but the evidence base was small and the included studies were considered at high risk of bias, reinforcing the need for cautious interpretation (106).

Direct evidence connecting exercise-induced immune alterations to protection from naturally acquired respiratory infection remains relatively limited. Importantly, however, some randomized prevention trials have assessed clinical respiratory illness rather than only biomarkers. In the original MEPARI trial, moderate exercise training was associated with trends toward lower acute respiratory illness burden and duration, although not all prespecified outcomes reached statistical significance (115). In the subsequent MEPARI-2 trial, moderate exercise showed a lower global severity of acute respiratory illness than the observational control group, but the prespecified significance threshold was not met for the primary analysis and the trial was considered potentially underpowered (115). Thus, clinical infection outcomes provide suggestive but not definitive evidence that regular moderate exercise may influence respiratory illness burden in some populations. Rises in vaccine antibody titers or changes in immune-cell trafficking should therefore not be automatically interpreted as lower susceptibility to infection.

### 8.4 Exercise, infection susceptibility, and context-dependent immune responses

The association between exercise and vulnerability to infection is likely to be context dependent rather than straightforwardly linear. Regular, moderate physical activity is generally linked to favorable or neutral immune-health profiles, while prolonged, high-volume, or unusually demanding exercise can be associated with temporary alterations in mucosal immunity, leukocyte trafficking, and inflammatory signaling. Nevertheless, these responses should not be taken as, by default, clinically significant immune suppression (69, 70). This differentiation matters because athletes engaged in intense training may simultaneously face sleep disruption, psychological stress, travel, energy shortfalls, environmental exposures, and other conditions that can independently affect susceptibility to infection (9, 108).

Separating acute from chronic responses is especially important. Acute physical activity can briefly change the distribution of immune cells and cytokine production, whereas repeated training may lead to adaptation to the same physiological stress. Thus, a transient post-exercise decline in a circulating immune-cell subset may reflect redistribution rather than loss of immune capacity, while a longer-term change in cellular phenotype may reflect adaptation without necessarily demonstrating improved host defense. In addition, exercise-related effects may be advantageous in one setting while remaining neutral or potentially unfavorable in another. For instance, increased leukocyte trafficking during exercise could facilitate immune surveillance, whereas subsequent changes during recovery may reflect redistribution or regulatory adaptation rather than impaired immune competence.

Psychological and behavioral factors may further shape this association. Among older adults, perceived stress, anxiety, and optimism were associated with influenza vaccine antibody responses, whereas exercise-related improvements in psychosocial indicators were proposed as potential contributors to changes in influenza-specific IFN-γ responses (71, 73). These observations support a multifactorial interpretation but do not establish mediation or demonstrate that psychosocial changes caused the immunological outcomes. They also highlight why interventions intended to influence respiratory infection outcomes should consider adherence, sleep, stress, behavioral engagement, and environmental exposure alongside biological mechanisms. Overall, the current body of evidence does not support a straightforward conclusion that engaging in more exercise necessarily yields stronger immune protection. Rather, host defense appears to depend on an interplay among exercise dose, recovery, baseline immune status, nutritional and metabolic state, environmental stressors, and the specific pathogen or immune challenge involved. Importantly, “exercise-associated immune change,” “immune competence,” and “clinical host defense” should be treated as distinct levels of evidence. This framework may help reconcile apparently contradictory findings across studies.

### 8.5 Age, training status, sex, and nutritional state

Age plays a central role in the immune responses elicited by exercise. In older adults, changes in adaptive immunity can be observed, including alterations in T-cell composition and reduced responsiveness to vaccination, potentially creating greater scope for behavioral interventions to affect selected immune outcomes. Some long-term exercise trials involving older adults have shown improved influenza vaccine responses (62, 73), whereas other randomized studies have not identified a significant vaccine benefit (71). This inconsistency suggests that age alone is insufficient to predict the immunological response to exercise and that baseline fitness, immune status, exercise modality and dose, and characteristics of the intervention must also be considered.

Training status can similarly affect acute immune responses. Older adults who are physically active versus inactive show distinct T-cell mobilization patterns after cardiorespiratory and resistance exercise (64). This suggests that the same exercise stimulus may not trigger the same immune response across different populations. Elite athletes offer another illustration: prolonged training, particularly when combined with environmental stressors such as hypoxia, may be associated with reductions in mucosal IgA (70). However, these athlete-specific observations should not be extrapolated directly to generally active adults or older populations. Taken together, these results highlight the need to differentiate physiological adaptation to regular exercise from immune responses associated with repeated high training volumes. Sex and endocrine status also warrant greater consideration. Immune responses differ between women and men, and hormonal changes across the life course, including menopause, may influence inflammatory signaling, immune-cell composition, substrate utilization, and responses to exercise and vaccination (19, 20). Accordingly, future exercise–immunity studies should report sex-stratified analyses where adequately powered and should consider menopausal status and other relevant endocrine factors rather than assuming that findings from predominantly male or mixed-sex cohorts are directly generalizable.

Nutritional and metabolic status can serve as additional effect modifiers of immune responses triggered by exercise. While the studies summarized here provide only restricted direct evidence regarding nutrition-specific modulation of host-defense outcomes, immune-cell function depends on energy and substrate availability, while exercise imposes additional metabolic demands. Energy availability, carbohydrate availability, protein intake, micronutrient status, adiposity, metabolic disease, and recovery nutrition may therefore alter the biological response to a given dose of exercise. Low carbohydrate availability should not be treated as equivalent to low energy availability, because they correspond to separate physiological states that may yield different immunometabolic outcomes. Among highly active individuals, inadequate energy availability and insufficient recovery may be especially important confounding factors or effect modifiers when interpreting immune biomarkers (92). Therefore, nutritional condition, energy availability, recovery, and training load should be considered when interpreting immune findings, especially in highly active groups. Finally, when interpreting associations between exercise and host-defense, it is important to take into account several potentially relevant confounders and effect modifiers, including obesity and visceral adiposity, type 2 diabetes and metabolic syndrome, medications, smoking, sleep duration and quality, psychological stress, circadian timing, recent infection or vaccination, habitual physical activity, cytomegalovirus serostatus, and gut microbiome composition. These factors may affect both exercise behavior and immune outcomes, thereby making causal inference more complex, especially in observational studies.

Taken as a whole, the evidence supports a framework in which exercise functions as a physiological modulator of host defense, with its effects shaped by the interplay among exercise dose, recovery, age, sex, training status, nutritional and metabolic environment, and baseline immune competence. Regular, sustainable exercise may be associated with preservation or favorable modulation of selected immune phenotypes, whereas extended-duration or high-intensity exercise performed under conditions of inadequate recovery may produce transient alterations in mucosal and cellular immune biomarkers. These patterns should not be interpreted as evidence of either universal immune enhancement or exercise-induced immunodeficiency. Notably, these mechanistic results should not be taken as evidence that exercise prevents or causes respiratory infections. The strongest translational priority is therefore to determine whether exercise-induced changes in immune biomarkers and cellular phenotypes translate into clinically relevant outcomes, including infection incidence, duration and severity, pathogen-specific immune responses, vaccine effectiveness, hospitalization, and recovery from infectious disease. Accordingly, future research should connect mechanistic biomarkers to clinically relevant outcomes while systematically accounting for exercise modality, intensity, duration, recovery, training status, age, sex, nutritional and metabolic conditions, environmental and psychosocial stressors, and the timing of immune challenges. Prospective studies should also distinguish acute redistribution from chronic adaptation and should predefine functional or clinical host-defense endpoints rather than relying solely on circulating biomarkers.

## 9 Integrating the exercise–myokine–mitochondria–nutrition–immunity axis

### 9.1 A unified mechanistic model

The available evidence supports a framework in which exercise-driven immunometabolic adaptation is best viewed as a coordinated, cross-tissue response rather than the downstream effect of a single molecular pathway. Skeletal-muscle contraction can alter the systemic environment through the release of cytokines and other exercise-responsive mediators, including IL-6 and IL-15, while simultaneously modifying substrate utilization and intracellular energy-sensing pathways (32, 116). Importantly, however, not all exercise-responsive mediators are exclusively muscle-derived, and muscle expression, secretion, circulating concentration, receptor engagement, and experimentally demonstrated immune-cell effects represent distinct biological levels. These events unfold in parallel with shifts in mitochondrial activity and cellular redox state, which together create multiple possible points of connection between exercise metabolism and immune control. Mitochondrial responses should likewise be interpreted at multiple levels, including biogenesis, respiratory function, redox signaling, mitochondrial dynamics, and mitophagy, because changes in a single mitochondrial marker do not establish increased mitochondrial content or completed mitochondrial quality control. The size and biological significance of these responses vary with exercise duration, intensity, training status, recovery, age, and nutritional conditions.

For instance, exercise-induced IL-6 expression in adipose tissue is reduced when carbohydrate is supplied, showing that substrate availability can influence endocrine signaling rather than acting only downstream of exercise (116). In humans, prolonged exercise increased adipose-tissue IL-6 gene expression, whereas carbohydrate ingestion substantially attenuated this response, demonstrating that nutritional availability can modify tissue-specific exercise signaling (116). This observation supports the concept of nutrition as a modifier of exercise signaling, but it does not establish that carbohydrate availability necessarily improves immune function or host defense.

Additional experimental findings indicate that IL-6 can participate in tissue-level metabolic responses to exercise. For example, pharmacological IL-6 receptor blockade abolished the exercise-associated reduction in visceral adipose tissue mass in abdominally obese adults, providing experimental evidence for a role of IL-6 signaling in a specific metabolic adaptation (117). This finding supports context-specific biological activity of IL-6 but does not establish IL-6 as a universal mediator of exercise adaptation or immune protection. In macrophages, exercise-related alterations in lipid mediators have likewise been experimentally associated with AMPK-dependent changes in mitochondrial respiration, offering a potential link between systemic exercise signals and immune-cell metabolism (51). However, this evidence is predominantly preclinical and mechanistic: exercise training altered specialized proresolving lipid mediators in mice, while human and murine macrophages exposed to selected mediators *in vitro* showed AMPK-dependent changes in mitochondrial respiration (51). Thus, these findings support biological plausibility for exercise–lipid mediator–mitochondrial interactions in macrophages but do not demonstrate that the same pathway operates *in vivo* in older humans or that it improves host defense.

Taken together, these observations are consistent with a distributed model in which muscle-derived and non-muscle-derived exercise-responsive signals, mitochondrial metabolism, nutrient availability, and immune-cell state interact in a dynamic manner. The proposed model should therefore be regarded as an integrative conceptual framework rather than a demonstrated linear pathway. Current evidence supports several individual connections among these components, whereas the simultaneous operation and relative contribution of the entire exercise–myokine–mitochondria–nutrition–immunity network in humans remain incompletely established. This distinction is particularly important because evidence obtained from muscle tissue, circulating biomarkers, isolated immune cells, animal models, and clinical outcomes represents different levels of biological inference.

### 9.2 Tissue–organ crosstalk in immunometabolic adaptation

A key implication of this framework is that immune adaptation cannot be inferred solely from measurements collected from immune cells circulating in the blood. Exercise alters skeletal muscle, adipose tissue, circulating metabolites, endocrine signals, and immune-cell bioenergetics at the same time, thereby enabling bidirectional communication between metabolically active tissues and the immune system. This cross-tissue model also includes potential contributions from liver, adipose tissue, vascular endothelium, bone, gut, and other organs that release exercise-responsive mediators. Consequently, the term “myokine” should not be interpreted as synonymous with every circulating exercise-responsive factor. Evidence from human studies indicates that exercise-related signals are not limited to contracting muscle. For instance, IL-6 expression rises in adipose tissue during prolonged exercise and is modulated by carbohydrate availability, implying that adipose tissue can contribute to the systemic response to exercise instead of serving only as a passive energy depot (118). However, the magnitude and direction of adipose IL-6 responses may vary according to depot, exercise duration, metabolic state, and experimental conditions. Thus, adipose tissue should be viewed as an active component of the exercise-responsive endocrine network rather than as a uniform source of IL-6.

In a randomized intervention, pharmacological blockade of IL-6 signaling reduced the exercise-associated loss of visceral adipose tissue mass, offering experimental support that IL-6 signaling can influence another metabolically relevant tissue (117). This study provides relatively direct human causal evidence for one exercise–IL-6–adipose tissue interaction, but it should not be extrapolated to immune-cell remodeling or to clinical host defense. On the level of immune cells, exercise has been associated with measurable changes in mitochondrial bioenergetics in circulating peripheral blood mononuclear cells (119). For example, acute maximal exercise in collegiate swimmers increased the number of circulating PBMCs and altered selected measures of tissue-level oxygen flux, whereas several intrinsic respiratory states did not change at the cellular level (49). This distinction illustrates why changes in whole-blood or PBMC bioenergetics should not automatically be interpreted as uniform metabolic reprogramming of immune cells. The composition of circulating immune cells changes markedly during and after exercise; as a result, bulk measurements from PBMCs can capture both intrinsic cellular adaptation and alterations in the relative proportions of distinct cell subsets. This issue is particularly important because exercise preferentially mobilizes specific lymphocyte populations, including differentiated CD8+ T-cell subsets. Accordingly, the translational relevance of tissue–immune communication depends on disentangling these cellular contributions.

Conversely, human observational evidence suggests that exercise training status may be associated with mitochondrial characteristics within specific immune-cell subsets. In active adults, naïve CD8+ T cells exhibited greater mitochondrial mass than in inactive adults, whereas mitochondrial ATP production, membrane potential, and biogenesis did not differ significantly between groups (120). This finding is informative but associative and does not demonstrate that exercise caused the mitochondrial difference or that greater mitochondrial mass translated into improved immune function. Therefore, evidence for tissue–immune communication should be interpreted hierarchically: (i) tissue-level exercise signaling; (ii) circulating mediator changes; (iii) immune-cell phenotype or bioenergetic changes; (iv) cell-specific functional responses; and finally (v) pathogen-specific or clinical host-defense outcomes. Evidence at one level should not be assumed to establish the next. Future studies integrating tissue-specific sampling, immune-cell phenotyping, mitochondrial flux measurements, and functional immune assays may be particularly valuable for differentiating circulating biomarkers from cell-intrinsic adaptations.

### 9.3 Interactions between exercise, nutrition, and immune aging

The interplay between physical activity and nutrition may be especially important as people age, since both metabolic reserve and immune-cell composition shift across the lifespan. In this framework, nutrition can be considered in at least four partially overlapping roles: (1) a direct determinant of energy and nutrient availability for immune-cell metabolism; (2) a modifier of exercise-induced signaling and mitochondrial adaptation; (3) a determinant of recovery and the ability to tolerate repeated exercise; and (4) a context-dependent factor whose effects may differ according to age, metabolic health, training load, and baseline nutritional status. The availability of nutrients can influence the magnitude of exercise-induced signaling, without necessarily determining whether a beneficial adaptive response develops.

In trained athletes, abundant carbohydrate intake during phases of higher exercise workload has been linked to cortisol profiles and salivary IgA responses that differ from those observed with a self-selected diet (52, 53). However, more recent periodized carbohydrate approaches have yielded comparatively small effects on selected immune markers (52, 53). Taken together, these results do not support a straightforward model in which greater carbohydrate availability consistently improves immune adaptation. Moreover, carbohydrate availability should not be equated with overall energy availability; short-term manipulation of carbohydrate intake and chronic low energy availability represent physiologically distinct conditions. Nutritional influences likely vary with training load, baseline nutritional status, timing of intake, recovery, and the specific immune outcome being assessed.

Dietary lipids may add an additional level of regulation. A recent randomized trial in physically trained adults reported that 12 weeks of DHA supplementation increased the Omega-3 Index and modified selected post-exercise inflammatory responses, although the study did not demonstrate a broad enhancement of immune function (60). More recent randomized evidence similarly indicates that 12 weeks of DHA supplementation in physically trained adults increased the Omega-3 Index and attenuated delayed-onset muscle soreness, while altering selected post-exercise IL-6, TNF-α, and IL-10 responses; these findings primarily support effects on recovery and inflammatory responses rather than direct immune-cell reprogramming (60). At the cellular level, experimental findings in macrophages indicate that exercise-associated changes in proresolving lipid mediators may affect mitochondrial respiration through AMPK-dependent pathways, offering a potential mechanistic connection among lipid metabolism, exercise signaling, and immune-cell metabolism (51). Because this evidence combines animal exercise models with *ex vivo* and *in vitro* macrophage experiments, it should be regarded as preclinical mechanistic evidence rather than direct evidence that dietary lipid supplementation reproduces this pathway in humans.

These considerations are especially pertinent to aging, in which metabolic flexibility, mitochondrial quality control, and immune-cell homeostasis may already be altered. Age-related changes in adiposity, insulin sensitivity, habitual activity, medication use, protein and micronutrient intake, sleep, and energy availability may further modify the response to exercise and nutrition. Even so, it remains unclear whether nutritional intervention can meaningfully reroute age-related immune trajectories in humans. Consequently, the existing body of evidence aligns more closely with nutrition functioning as a context-dependent modifier of exercise-driven remodeling than with nutrition serving as an independent catalyst for reversal of immune aging.

Importantly, the available evidence does not justify assuming that combining exercise with a particular nutrient produces additive or synergistic immune benefits unless the interaction has been directly tested. In many cases, nutrition and exercise have been studied separately, or the outcomes have been limited to metabolic, inflammatory, or recovery-related biomarkers. Future factorial or mechanistically controlled trials will be needed to determine whether specific nutritional conditions modify exercise-induced immune adaptations in a reproducible and clinically relevant manner.

### 9.4 From molecular reprogramming to host defense

The last translational step is to assess whether exercise-associated molecular and metabolic remodeling results in changes to host defense that are functionally meaningful. This distinction matters because shifts in cytokine concentrations, mitochondrial respiration, immune-cell phenotype, or metabolic gene expression do not automatically correspond to greater resistance to infection or to more effective immune surveillance. Studies in humans show that exercise can alter measurable features of immune cells, including mitochondrial characteristics of naïve T cells and the bioenergetic responses of circulating mononuclear cells (119, 120). However, these findings remain intermediate biological outcomes. For example, greater mitochondrial mass in naïve CD8+ T cells was associated with aerobic fitness, whereas acute exercise-related changes in PBMC bioenergetics were influenced by changes in circulating cell numbers (49, 120). Neither study established improved antigen-specific immunity, vaccine effectiveness, resistance to infection, or clinical host defense.

Likewise, experimental work has shown that exercise-related changes in macrophage metabolism and inflammatory responsiveness occur, yet it remains unclear to what degree these cellular alterations affect host defense in humans (51). The macrophage findings therefore provide mechanistic plausibility rather than direct evidence of improved antimicrobial protection in exercising humans.

Aging introduces an additional level of complexity. A recent three-year longitudinal study reported that declining physical fitness was associated with multiple aspects of immune remodeling in otherwise healthy older adults, whereas classical circulating inflammatory cytokines remained relatively stable (103). Specifically, the study observed reductions in naïve and regulatory T-cell populations alongside increases in differentiated and senescence-associated T-cell subsets, while serum concentrations of several classical pro-inflammatory cytokines remained largely stable (103). These findings reinforce the distinction between immune-cell remodeling and systemic inflammaging and suggest that cellular immune aging may progress even when conventional inflammatory biomarkers show limited change. Because the study was longitudinal observational rather than randomized, however, it does not establish that changes in physical fitness caused the observed immune remodeling. This finding underscores the need for caution when using inflammatory biomarkers alone as stand-ins for immune resilience. More broadly, the translational evidence can be organized into a hierarchy: molecular biomarkers → immune-cell phenotype → cellular functional responses → vaccine-specific responses → naturally acquired infection outcomes → clinically meaningful outcomes such as infection severity, duration, hospitalization, or recovery. Evidence that remains at an upstream level should not be interpreted as proof of downstream clinical benefit.

The connection between molecular adaptation and host defense is likely to vary by immune-cell subset, pathogen exposure, vaccination status, nutritional state, metabolic health, sex, age, CMV serostatus, training history, and recovery. These factors may act as effect modifiers rather than merely background characteristics and should therefore be incorporated into study design and analysis. In observational studies, reverse causation and healthy-participant bias should also be considered because better baseline health, metabolic status, and functional capacity may independently favor both greater exercise participation and more favorable immune profiles.

Therefore, the most defensible translational interpretation is not that exercise universally restores immune competence, but rather that repeated physical activity may modify several biological conditions relevant to immune resilience. Determining whether these adaptations translate into meaningful preservation of host defense will require sufficiently powered prospective and, where feasible, randomized studies that simultaneously assess exercise dose, nutritional context, immune-cell subsets, mitochondrial function, vaccine responses, and clinically relevant infection outcomes. Such studies should distinguish acute redistribution from chronic adaptation and should avoid treating circulating biomarkers as surrogate measures of immune competence without validation.

Accordingly, **Figure 2** presents a graded evidence model rather than a single causal pathway: solid arrows represent relationships supported by direct mechanistic evidence, whereas dashed arrows indicate indirect, extrapolated, or hypothesized connections. Evidence derived from human intervention studies, human associative studies, animal/preclinical experiments, and *in vitro* systems is explicitly differentiated. This representation is intended to communicate the central contribution of the review—an integrated conceptual framework linking exercise, myokine/exerkine signaling, mitochondrial adaptation, nutritional context, and immune aging—without implying that the complete axis has been causally demonstrated in humans.

**Figure 2:**
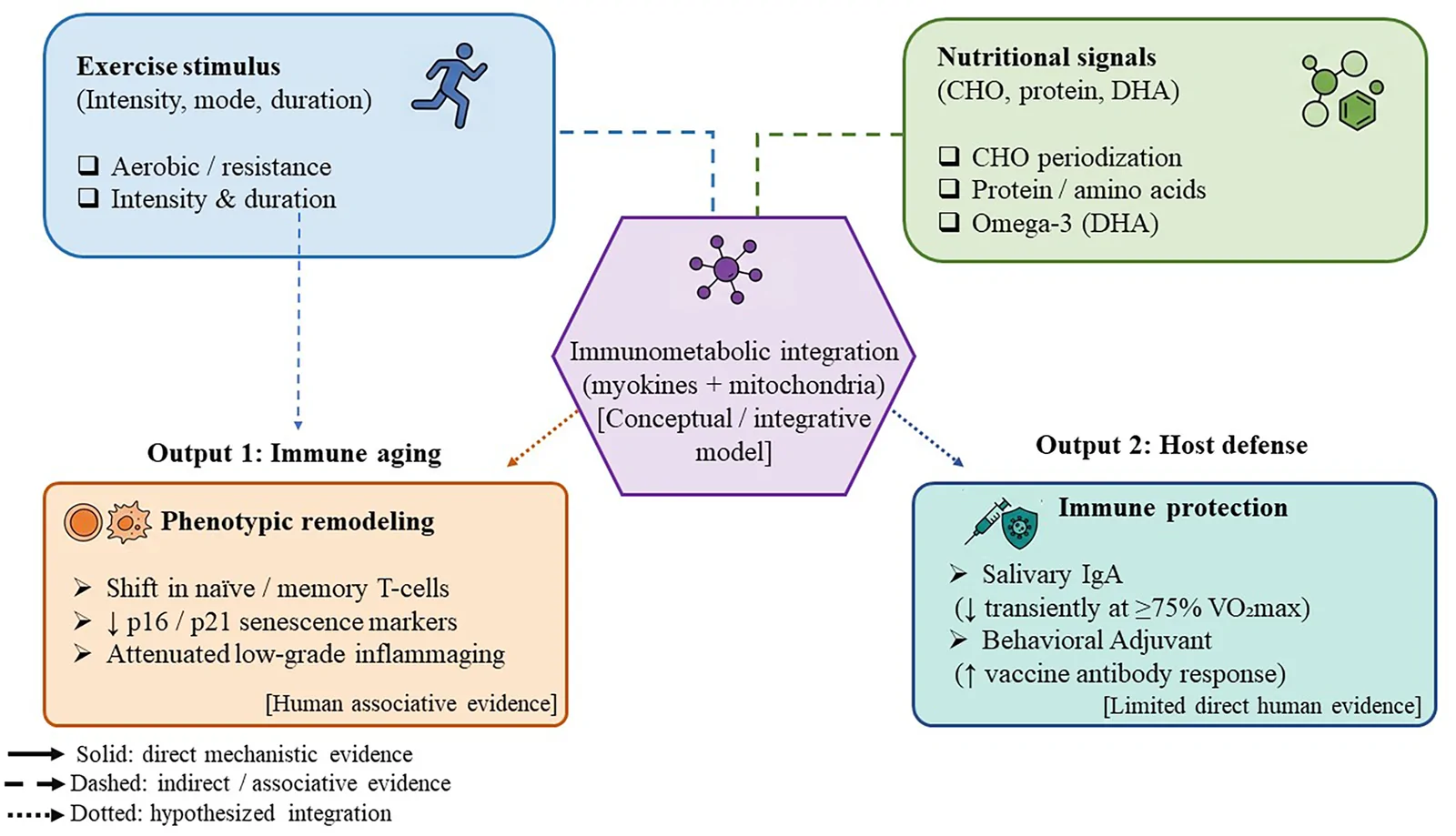
Integrative exercise–nutrition framework for immunometabolic remodeling, immune aging, and host defense. Exercise-related stimuli and nutritional factors converge within a context-dependent immunometabolic network involving skeletal muscle, metabolic tissues, mitochondria, and immune cells. Exercise modality, intensity, duration, training status, age, sex and endocrine status, nutritional and energy availability, metabolic health, and other biological and environmental factors may modify the magnitude and direction of these responses. The framework links exercise and nutritional inputs with molecular biomarkers, immune-cell phenotypes and function, mitochondrial adaptations, vaccine-related immune responses, and ultimately pathogen- and clinically relevant host-defense outcomes. Changes in markers associated with immune aging, including naïve-to-memory T-cell remodeling or expression of cellular-senescence-related markers such as p16 and p21, are presented as evidence of selected aspects of immune remodeling rather than as proof of reversal of immunosenescence. Likewise, enhanced antibody responses following vaccination represent a functional immune endpoint but do not by themselves establish protection against infection or clinical disease. Solid arrows represent direct or relatively well-supported relationships, whereas dashed arrows indicate indirect, extrapolated, associative, or hypothesized connections. Human intervention and observational evidence are distinguished from animal/preclinical and *in vitro* evidence, and molecular or cellular findings are not interpreted as equivalent to clinical host-defense outcomes. Thus, the figure represents an integrative conceptual framework for interpreting how exercise and nutrition may shape immunometabolic remodeling during aging, rather than a single causally established pathway or a uniformly beneficial model of immune protection.

## 10 Clinical and translational perspectives

The increasing interest in exercise as a modifiable factor for immune and metabolic health creates multiple opportunities for clinical investigation. Instead of treating exercise only as a broad lifestyle recommendation, an immunometabolic viewpoint could help inform the design of approaches that are more grounded in biology for distinct populations. Nevertheless, moving from mechanistic findings to clinical practice still depends on the consistency, reproducibility, standardization, safety, feasibility, and clinical relevance of the existing evidence. A plausible future application involves integrating immunometabolic indicators into exercise and healthy-aging interventions. Circulating myokines, inflammatory mediators, metabolic markers, mitochondrial-related signatures, and immune phenotypes may serve as complementary biomarkers to characterize how individuals respond to physical activity. However, these measurements should currently be regarded primarily as research and stratification tools rather than established clinical decision-making instruments. Importantly, changes in circulating biomarkers, mitochondrial-related signatures, or immune-cell phenotypes should not be interpreted as evidence of improved immune competence unless accompanied by functional immune or clinically meaningful outcomes. Many proposed biomarkers have not yet achieved sufficient standardization and validation for routine clinical application. In addition, their interpretation may be shaped by age, sex, menopausal or endocrine status, medication use, baseline fitness, nutritional status, sleep, adiposity, metabolic health, habitual physical activity, recent infection or vaccination, and recent exposure to exercise.

Personalized exercise prescription may therefore become increasingly pertinent as a research direction. Exercise frequency, intensity, duration, and modality could potentially be tailored to individual metabolic and immune profiles rather than depending solely on broadly generalized guidance. However, personalized exercise prescription based specifically on immunometabolic phenotyping should not yet be considered an established clinical strategy, because validated biomarkers, response thresholds, and prospective evidence linking phenotype-guided exercise selection to patient-centered outcomes remain insufficient. Nutritional approaches may add an extra dimension of personalization, especially when dietary intake, energy availability, or nutrient deficiencies alter physiological responses to training. Such nutritional factors may function not only as potential modifiers of exercise signaling but also as determinants of substrate availability, recovery, and the interpretation of immunometabolic responses. Even so, the evidence for particular exercise–nutrition pairings in relation to immune-related clinical outcomes remains inconsistent, and no definitive therapeutic protocols can yet be established from mechanistic findings alone.

Clinical translation should also distinguish several levels of outcome. Molecular biomarkers and circulating mediators represent an early level of evidence, followed by cellular functional responses, antigen- or vaccine-specific immune responses, pathogen-related outcomes, and ultimately patient-centered clinical outcomes such as infection incidence, severity, duration, hospitalization, or recovery. Evidence at one level should not automatically be interpreted as evidence at the next. Collaboration across exercise physiology, immunology, nutrition, gerontology, and infectious disease research may also enhance translation efforts. Prospective clinical studies employing standardized exercise protocols and harmonized nutritional assessments, adequately characterized participant populations, and immune outcomes that are clinically meaningful will be crucial for assessing whether the molecular changes observed following exercise translate into measurable benefits for health or resilience. Such studies should explicitly account for major sources of heterogeneity and confounding, including age, sex and menopausal status, adiposity and metabolic disease, medication use, smoking, sleep, psychological stress, circadian timing, habitual activity, energy availability, recent infection or vaccination, and other relevant lifestyle or biological factors. Ultimately, the clinical value of exercise-induced immunometabolic remodeling will depend not only on biological plausibility but also on its reproducibility, safety, feasibility, effectiveness in well-defined populations, and demonstrable relevance to patient-centered outcomes.

## 11 Future directions

Several areas need additional investigation before the biological significance of exercise-driven immunometabolic remodeling can be determined with greater precision. A key priority is to develop longitudinal studies that can differentiate short-lived responses to single exercise sessions from enduring adaptations produced by long-term training. Studies should explicitly separate acute responses from chronic adaptations and characterize exercise according to modality, intensity, duration, frequency, recovery, and training status rather than treating “exercise” as a uniform exposure. Collecting repeated samples before, during, and after exercise interventions may help elucidate the timing relationships among metabolic signals, immune cell phenotypes, and functional outcomes. Repeated sampling should ideally be combined with analyses of defined immune-cell subsets, because changes in whole-blood or peripheral blood mononuclear cell measurements may partly reflect exercise-induced redistribution of immune-cell populations rather than intrinsic cellular adaptation.

Multi-omics approaches are another promising avenue. Integrating transcriptomics, proteomics, metabolomics, lipidomics, epigenomics, and mitochondrial profiling could enable a more complete characterization of individual responses to exercise. Such methods may also assist in identifying molecular signatures linked to distinct training adaptations. However, discovery of a molecular signature should not be equated with demonstration of biological function or clinical utility. Importantly, discovery-based studies should be followed by independent validation cohorts to assess whether proposed signatures are reproducible and clinically relevant. Future studies should also establish whether candidate biomarkers predict functional immune responses or clinically meaningful outcomes rather than merely correlate with exercise exposure or physiological adaptation.

Future research should more explicitly incorporate biological heterogeneity. Variability in exercise-related immune responses may be shaped by factors including age, sex, menopausal or endocrine status, genetic background, adiposity, baseline physical fitness, medication exposure, dietary patterns, energy availability, sleep, circadian biology, and the gut microbiome. Differences between younger and older adults, athletes and sedentary individuals, and metabolically healthy and metabolically compromised populations should be considered explicitly rather than pooled without adequate justification. Sex-stratified analyses and sufficient representation of women, including consideration of menopause and endocrine aging, may be particularly informative for determining whether apparently inconsistent responses reflect meaningful biological heterogeneity. Stratified studies with sufficient statistical power could clarify whether findings that appear inconsistent actually stem from true biological differences across populations. Observational studies should additionally address healthy-participant bias and reverse causation, particularly when higher fitness is associated with more favorable immune or metabolic profiles.

Further attention is warranted for the interaction between exercise and infection-related outcomes. Instead of presuming a single, uniform association between physical activity and host protection, future studies should test how exercise dose, recovery, nutritional availability, age, sex, immune status, and pathogen characteristics affect immune responses under well-defined conditions. Controlled experimental models may also complement prospective human studies in this domain. Nevertheless, mechanistic findings from experimental models should be used to generate and test hypotheses rather than being presented as direct evidence of clinical protection in humans.

Finally, future investigations should progress beyond biomarker discovery toward functional and clinically meaningful endpoints. This progression should include, where appropriate, validated measures of immune-cell function, antigen-specific or vaccine responses, pathogen-specific immune responses, infection incidence and severity, duration of illness, hospitalization, and other patient-centered outcomes. Establishing standardized definitions of immune resilience, using validated immunometabolic biomarkers, implementing reproducible exercise interventions, and conducting harmonized nutritional assessments would improve the comparability of results across studies. Methodological harmonization should also include standardized reporting of exercise dose, timing of biological sampling, nutritional status, medication use, sex and menopausal status, and assay characteristics, particularly for circulating myokines and mitochondrial measurements. Combining mechanistic experimentation with longitudinal clinical research may ultimately clarify which components of exercise-induced immunometabolic remodeling are merely associated with adaptation and which, if any, have meaningful implications for healthy aging or disease-related outcomes. An important priority will be to determine whether observed changes represent transient physiological responses, durable adaptations, or clinically relevant preservation of immune function during aging.

## 12 Conclusions

Exercise-induced immunometabolic remodeling is a multifaceted biological process that entails signaling between skeletal muscle, metabolic systems, mitochondria, nutritional inputs, and immune networks. In this review, these elements are integrated within a framework that evaluates their potential significance for immune aging and host defense. The current body of literature indicates that exercise can modulate numerous molecular and cellular mechanisms that participate in immune regulation; however, the size, direction, and durability of these effects may vary substantially according to biological and contextual conditions. The idea of immunometabolic reprogramming may offer a valuable lens for explaining why exercise effects cannot be understood solely as changes in inflammatory biomarkers or as alterations in individual immune cell subsets. Rather, metabolic status, cellular energy pathways, communication across organs, and nutritional context may jointly shape the immune outcomes of physical activity. These relationships should nevertheless be interpreted as a context-dependent network rather than as a single linear or uniformly beneficial pathway. This integrated perspective may be especially pertinent for older populations, for whom physiological reserve and reactivity to environmental stimuli can differ markedly.

At the same time, considerable uncertainty persists about how molecular adaptations translate into outcomes that are clinically meaningful. Findings from cellular and animal models cannot always be directly generalized to humans, and human studies often vary in exercise protocols, participant characteristics, nutritional status, and methods of outcome assessment. Moreover, human evidence spans molecular biomarkers, circulating mediators, immune-cell phenotypes, cellular bioenergetics, and functional immune outcomes, and these levels of evidence should not be considered interchangeable. In particular, an increase in a myokine, a change in inflammatory biomarkers, enhanced mitochondrial signaling, or altered immune-cell phenotype does not by itself establish improved immune competence, vaccine effectiveness, infection resistance, or other clinical benefit. Therefore, links between exercise-associated molecular signatures and immune competence should not be taken as proof of direct causation without adequate support from longitudinal or intervention-based research.

The evidence synthesized in this review also indicates that exercise-associated immunometabolic remodeling is unlikely to be uniform across individuals. Age, sex and endocrine status, training status, exercise modality and dose, adiposity and metabolic health, nutritional and energy availability, sleep, circadian factors, medication exposure, recent infection or vaccination, and other biological and environmental conditions may modify observed responses. Accordingly, personalized exercise and nutrition strategies based on immunometabolic phenotyping represent promising areas for future investigation rather than established clinical approaches.

Overall, exercise may be considered one element within a wider immunometabolic milieu, rather than an isolated determinant of immune function. Its potential relevance to immune aging should therefore be considered in relation to nutrition, metabolic health, biological aging, tissue–organ communication, and environmental context. Clarifying how exercise interacts with these interacting determinants will require studies that integrate mechanistic measurements with functional immune and clinically meaningful endpoints. Ongoing integration of mechanistic, longitudinal, and translational evidence will be needed to establish which exercise-induced adaptations are reproducible, biologically significant, functionally relevant, and potentially suitable for supporting immune health throughout the lifespan. Importantly, the present structured narrative framework is intended to organize the available evidence and identify testable mechanistic relationships, not to imply that a complete exercise–myokine–mitochondria–nutrition–immunity pathway has been causally established in humans.
