# Supplementary material for: Exercise–nutrition–myokine–mitochondrial interactions in immune aging: an immunometabolic framework for host defense
Source: Front Immunol. 2026 Oct 30;17:1987314. doi: 10.3389/fimmu.2026.1987314 (PMC13760681; doi:10.3389/fimmu.2026.1987314)
Supplement: Supplementary file 1 [file Table1.docx]

**Supplementary Table S1.**

Database-specific and supplementary search strategies used to identify literature on exercise-induced immunometabolic reprogramming, myokine/exerkine signaling, mitochondrial adaptation, nutritional modulation, immune aging, inflammation, and host defense.

*Article: Exercise–nutrition–myokine–mitochondrial interactions in immune aging: an immunometabolic framework for host defense. Corresponding author: Junpeng Pang (Wuhan Sports University).*

| **Database** | **Search platform / field** | **Search date** | **Search strategy / representative search string** | **Limits / notes** |
| --- | --- | --- | --- | --- |
| PubMed/MEDLINE | Title/Abstract; MeSH terms where applicable | July 2026 | (("Exercise"[Mesh] OR "Exercise Therapy"[Mesh] OR exercise[Title/Abstract] OR "physical activity"[Title/Abstract] OR "exercise training"[Title/Abstract] OR "aerobic exercise"[Title/Abstract] OR "resistance exercise"[Title/Abstract] OR "interval training"[Title/Abstract]) AND ("Immunometabolism"[Title/Abstract] OR "immune metabolism"[Title/Abstract] OR "immune function"[Title/Abstract] OR "immune regulation"[Title/Abstract] OR "host defense"[Title/Abstract] OR inflammation[Title/Abstract] OR "immune aging"[Title/Abstract] OR immunosenescence[Title/Abstract] OR inflammaging[Title/Abstract]) AND (myokine*[Title/Abstract] OR exerkine*[Title/Abstract] OR "skeletal muscle"[Title/Abstract] OR "muscle-derived"[Title/Abstract] OR "mitochondrial adaptation"[Title/Abstract] OR "mitochondrial biogenesis"[Title/Abstract] OR "mitochondrial function"[Title/Abstract] OR mitophagy[Title/Abstract]) AND (nutrition*[Title/Abstract] OR "nutritional modulation"[Title/Abstract] OR protein*[Title/Abstract] OR "amino acid*"[Title/Abstract] OR micronutrient*[Title/Abstract] OR diet*[Title/Abstract] OR supplement*[Title/Abstract])) | No restriction on full-text language; English-language abstract required for eligibility. Database inception to 31 July 2026. MeSH terms and free-text terms were combined. Additional targeted searches were conducted for vaccination, infection, antimicrobial/antiviral immunity, and specific myokines/exerkines when required during screening. |
| Web of Science Core Collection | Topic (TS) | July 2026 | TS=(exercise OR "physical activity" OR "exercise training" OR "aerobic exercise" OR "resistance exercise" OR "interval training") AND TS=(immunometabolism OR "immune metabolism" OR "immune function" OR "immune regulation" OR "host defense" OR inflammation OR "immune aging" OR immunosenescence OR inflammaging) AND TS=(myokine OR exerkine* OR "skeletal muscle" OR "muscle-derived" OR "mitochondrial adaptation" OR "mitochondrial biogenesis" OR "mitochondrial function" OR mitophagy) AND TS=(nutrition* OR "nutritional modulation" OR protein* OR "amino acid*" OR micronutrient* OR diet* OR supplement*)* | No restriction on full-text language; English-language abstract required for eligibility. Database inception to 31 July 2026. Topic searching included title, abstract, author keywords, and Keywords Plus. Reference-list screening of key studies and reviews was also performed. |
| Scopus | TITLE-ABS-KEY | July 2026 | TITLE-ABS-KEY (exercise OR "physical activity" OR "exercise training" OR "aerobic exercise" OR "resistance exercise" OR "interval training") AND TITLE-ABS-KEY (immunometabolism OR "immune metabolism" OR "immune function" OR immun OR "immune regulation" OR "host defense" OR inflammation OR "immune aging" OR immunosenescence OR inflammaging) AND TITLE-ABS-KEY (myokine OR exerkine* OR "skeletal muscle" OR "muscle-derived" OR "mitochondrial adaptation" OR "mitochondrial biogenesis" OR "mitochondrial function" OR mitophagy OR "redox signaling" OR "metabolic flexibility") AND TITLE-ABS-KEY (nutrition* OR "nutritional modulation" OR protein* OR "amino acid*" OR "fatty acid*" OR micronutrient* OR diet* OR supplement*)** | No restriction on full-text language; English-language abstract required for eligibility. Database inception to 31 July 2026. Search was performed in title, abstract, and author-keyword fields. Additional searches were used for specific exercise modalities, myokines, mitochondrial pathways, nutritional factors, vaccination, and host-defense outcomes. |
| Google Scholar | General keyword search | July 2026 | "exercise" "immunometabolism" myokine mitochondria nutrition | No language restriction. Used primarily as a supplementary source to identify relevant interdisciplinary, recent, or difficult-to-index publications and to screen cited/citing literature. Results were assessed for conceptual and methodological relevance rather than treated as an independent exhaustive database. |
| Reference-list screening | Backward citation searching | July 2026 | Reference lists of key primary studies and relevant high-quality reviews were manually screened for additional publications addressing exercise–immune interactions, myokines/exerkines, mitochondrial adaptation, nutritional modulation, immune aging, inflammation, vaccination, and host defense. | Used to identify potentially relevant publications not retrieved through the electronic database searches. |
| Citation tracking | Forward citation searching | July 2026 | Cited-by records of key mechanistic and clinical studies were examined selectively to identify subsequent human, animal, and cellular evidence relevant to the conceptual framework. | Used selectively to identify emerging evidence and clarify the development of important mechanistic concepts. |

Abbreviations: MeSH, Medical Subject Headings; TS, Topic; TITLE-ABS-KEY, title, abstract, and keywords.
